# An _e_pH-driven DNA nanodevice for impeding metastasis *in vivo* by selectively blocking cell signaling

**DOI:** 10.1093/nsr/nwae471

**Published:** 2025-01-07

**Authors:** Kun Yuan, Hong-Min Meng, Hongzhi Sun, Lingbo Qu, Zhaohui Li, Weihong Tan

**Affiliations:** College of Chemistry, Institute of Analytical Chemistry for Life Science, Zhengzhou University, Zhengzhou 450001, China; College of Chemistry, Institute of Analytical Chemistry for Life Science, Zhengzhou University, Zhengzhou 450001, China; College of Chemistry, Institute of Analytical Chemistry for Life Science, Zhengzhou University, Zhengzhou 450001, China; College of Chemistry, Institute of Analytical Chemistry for Life Science, Zhengzhou University, Zhengzhou 450001, China; College of Chemistry, Institute of Analytical Chemistry for Life Science, Zhengzhou University, Zhengzhou 450001, China; The First Affiliated Hospital of Zhengzhou University, Zhengzhou University, Zhengzhou 450052, China; Zhejiang Cancer Hospital, Hangzhou Institute of Medicine (HIM), Chinese Academy of Sciences, Hangzhou 310022, China; Institute of Molecular Medicine (IMM), Renji Hospital, Shanghai Jiao Tong University School of Medicine, and College of Chemistry and Chemical Engineering, Shanghai Jiao Tong University, Shanghai 200240, China

**Keywords:** antimetastatic therapy, cellular signaling blockade, DNA nanodevice, extracellular pH, tailored bioregulation

## Abstract

Invasion and metastasis dominate tumor progression, causing a substantial proportion of cancer-related deaths. However, the efficacy of current antimetastatic treatments is hampered by the dearth of targeted therapeutics. Recently developed synthetic-receptor toolkits offer potential for artificially regulating cellular behavior. However, to the best of our knowledge, none has yet successfully suppressed tumor metastasis *in vivo*. Here, we report the first extracellular pH (_e_pH)-driven DNA nanodevice for use in antimetastatic treatment *in vivo* by manipulating heterogeneous receptors on the tumor cell surface. This DNA nanodevice was constructed by partially locking tumorigenic receptor-specific aptamers with two i-motifs. Acidic extracellular pH induced dynamic allosteric reassembly within the nanodevice. The restructured nanodevice enabled oligomerization of c-Met and transferrin receptor, which inhibited tumor metastasis by blocking the hepatic growth factor (HGF)/c-Met signaling pathway. A suppressive efficacy of 86.25% was verified in an early hepatocarcinoma-pulmonary-metastasis mouse model. Such impressive antimetastatic efficacy suggests an efficient paradigm for developing adaptive antimetastatic therapeutics.

## Teaser

An extracellular pH-actuated DNA nanodevice was developed as an antimetastatic inhibitor to suppress tumor metastasis in vivo by selectively blocking hyperactive receptor signaling.

## INTRODUCTION

Metastasis, especially systemic invasion by small unvascularized micrometastasis [1–3], is typically incurable by local surgery or radiotherapy and causes ∼95% of cancer deaths [4–6]. Deep insights into the biological mechanisms of tumorigenesis reveal that metastasis relies on a multistep mechanochemical process governed by various receptors [7–9]. In particular, c-Met, a hyperactive malignant receptor that assembles into homodimers with hepatic growth factor (HGF) as ligands [10], acts as an effector for tumor cells to weaken adhesion within primary sites [11], drive detached cells to migrate [12] and ultimately trigger invasion and metastasis [13]. Therefore, remodeling the function of migration-related receptors offers the possibility of developing effective methods for antimetastatic therapy [14].

With the merits of flexible addressability and superior programmability [15–17], DNA nanotechnology emerged as an attractive candidate for tailored bioregulation [18–20]. For instance, Sando's group designed aptamer-based synthetic switches that reproduce the growth factor-induced activation process of c-Met [21]. This concept suggests that receptor activities could be manipulated through different molecular interaction patterns. Li's group reported a nongenetic method for logic modulation of endogenous receptor signaling, demonstrating the potential for Boolean regulation of receptor functions based on multiple inputs [22]. More recently, Nie's group reported a DNA-based artificial mechanoreceptor that reprograms non-mechanoresponsive c-Met with *de novo* force-triggered signaling transduction [23]. Such attempts show promise for artificially modulating receptor interactions at micro-/nano-scales by dynamic DNA nanotechnology [24–26]. Nevertheless, the potential of these strategies in living animals, particularly for treatment of metastatic tumors, remains to be exploited. Previous research revealed that aptamers could be designed as nucleic acid nanodrugs for tumor therapy by interrupting the functions of targeted proteins [27,28]. However, their therapeutic efficacy was found to be compromised by ‘on-target off-tumor’ effects in normal tissues owing to the substantial overlap in receptor profiles between metastatic tumor and naïve tissues [29]. Therefore, an effective solution to the prevailing dilemma is to endow prototypical DNA nanodevices with refreshed antimetastatic properties and dexterities to meet the urgent needs of precision medicine.

**Scheme 1.**
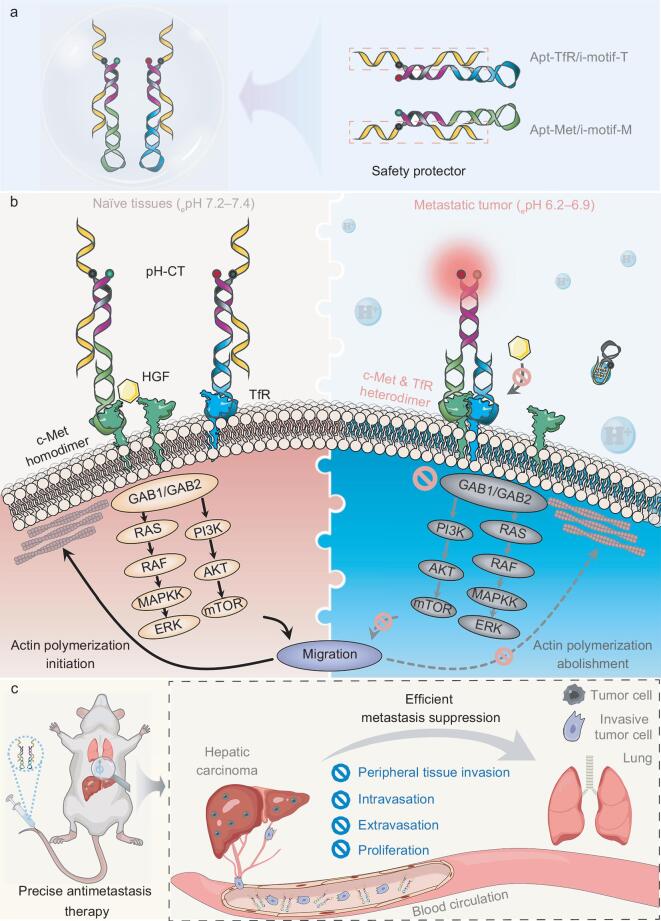
Schematic illustration of the *in vivo* antimetastatic effect of pH-CT. (a) Molecular structure of pH-CT. (b) Selective interception of metastasis-related c-Met/HGF signal transduction by pH-CT. (c) Precise suppression of pulmonary metastasis by pH-CT.

Cancer progression implicates a cascade of physicochemical alterations that result in reshaping the tumor microenvironment (TME) [30], which exhibits characteristics distinct from those of normal tissues [31]. By integrating features of the TME into the engineering of artificial receptors, it is possible to alleviate adverse toxicity. As a proof-of-concept, we exploited the acidic extracellular pH [32] within the TME and developed an _e_pH-activated DNA nanodevice, termed as pH-CT, to inhibit tumor metastasis by selectively interrupting the relevant signal transduction. As presented in Scheme 1, pH-CT was prepared by controlled assembly of two subcomponents, Apt-Met/i-motif-M and Apt-TfR/i-motif-T. Within metastatic tumors, pH-CT sensed the peripheral dysregulated pH through allosteric i-motif [33], enforcing the dynamic reassembly of two subcomponents and facilitating heterodimerization of c-Met and transferrin receptor (TfR). The resulting receptor-heterodimer inhibited c-Met function by blocking HGF interactions, ultimately suppressing tumor metastasis. Here, i-motif functioned as the *de facto* protector of healthy cells against pH-CT manipulation. Based on this sense-allostery-reassembly action, the antimetastatic efficacy of pH-CT was exclusively confined to c-Met-hyperactivated tumor cells, eliminating unwanted effects and realizing the goal of precise and efficient metastasis inhibition.

## RESULTS AND DISSCUSSION

### Design and implementation of pH-CT

**Figure 1.**
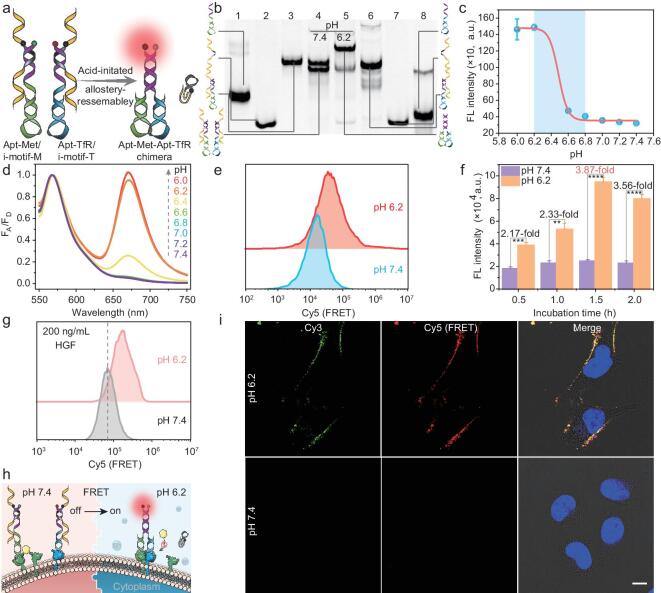
Fabrication and characterization of pH-CT. (a) Schematic illustration of _e_pH-driven self-allostery and reassembly of pH-CT. (b) Analysis of the designed pH-CT by PAGE. Lanes 1–8: Apt-Met, i-motif-M, Apt-Met/i-motif-M, pH-CT (pH 7.4), pH-CT (pH 6.2), Apt-TfR/i-motif-T, i-motif-T, and Apt-TfR. (c) Plot of FRET intensity of pH-CT versus pH values. (d) Relationship between Cy5/Cy3 fluorescence ratio and different pH values. (e, f) Flow cytometry (e) and quantification (f) of FRET signal of HepG-2 cells induced by pH-CT at pH 7.4 or 6.2 (*n* = 3). (g) Potential of pH-CT to induce receptor heterodimerization under HGF interference. (h) Schematic illustration of acidosis-induced heterogeneous protein dimerization by pH-CT. (i) Confocal fluorescence images of HepG-2 cells treated with pH-CT under different pH. Scale bars: 10 µm. Statistical significance was calculated by two-tailed Student's *t*-test: **, *P* < 0.01; ***, *P* < 0.001; ****, *P* < 0.0001.

To achieve a TME-activated DNA nanodevice, i-motif was introduced as the safety protector. Accordingly, two subcomponents were designed by hybridizing the fluorophore-labeled receptor-specific aptamers Apt-Met and Apt-TfR, containing an 18-base tail (purple) at the 3′-end, with their complementary BHQ2-modified i-motifs (yellow and grey, Fig. 1a). In the absence of acidic stimulation, the tails were sequestered in the Apt/i-motif duplexes, ensuring the inertness of spontaneous self-assembly of the two subcomponents. However, upon activation at acidic _e_pH, the embedded i-motifs folded into an H^+^-mediated quadruplex and were subsequently released from each subcomponent. Such reduced constraint led to the formation of c-Met/TfR aptamer chimeras via tail hybridization, organizing two anchored c-Met and TfR in close proximity.

### The performance of pH-CT under acidic _e_pH

The feasibility of pH-CT was first evaluated by 9% polyacrylamide gel electrophoresis (PAGE). As shown in Fig. 1b, following stabilization of i-motif at pH 7.4, pH-CT failed to undergo configuration transformation and reassembly, yielding two independent bands with different gradients (lane 4). Conversely, along with the assembly of c-Met/TfR aptamer chimera at pH 6.2, pH-CT showed the slowest gel migration (lane 5). This suggested successful activation of self-allostery and reassembly of pH-CT in an acidic environment. The acidic _e_pH-responsive allostery of pH-CT was then investigated by monitoring the Förster resonance energy transfer (FRET) signal generated between Cy3 and Cy5 under different pH milieux. By rigorous Watson–Crick hybridization, pH-CT displayed a negligible FRET signal at pH 7.4, indicating that the i-motif moiety functioned as a safety protector to maintain the inert state of pH-CT (Fig. S1 in Supplementary data). On the other hand, lowering the pH from 6.8 to 6.2 resulted in shifting the equilibrium away from i-motif sequestration, forming a c-Met/TfR aptamer duplex to produce an acutely enhanced FRET response (Fig. 1c). With a transition midpoint of pH 6.49 ± 0.14, such 0.6 pH-unit feedback lapsed into the dysregulated fluctuation of mild acidosis [34], suggesting that pH-CT was even sensitive to marginally acidic TME. Additionally, an auxiliary with the same structure as pH-CT, but without quencher labeling on the i-motifs, was designed to explore the FRET ratio of pH-CT. A 20.4-fold increment in F_Cy5_/F_Cy3_ ratio (from 0.05 to 1.02) was achieved when pH was reduced from 7.4 to 6.2 (Fig. 1d and Figs S2, S3). These results showed that pH-CT had gained sense-allostery-reassembly capability in an _e_pH-dependent manner, thus establishing the underpinning for the remodeling of tumor cell fate.

### Characterization of pH-CT in sense-and-transmit acidic TME input

Having established the pH-dependent reconfiguration property of pH-CT, we asked if it would induce heterodimerization between c-Met and TfR on the cell membrane in acidic TME. To make this determination, HepG-2 cells were selected for study because of their positive expression of these receptors (Fig. S4). Because of the high binding affinity of overhanging aptamers, pH-CT was tethered on the cell membrane, forming two DNA-chimeric artificial receptors (Fig. S5). Flow cytometry demonstrated that pH-CT induced a higher FRET signal on cell membranes at pH 6.2 than at pH 7.4 (Fig. 1e), indicating that pH-CT acted as a molecular mediator to dimerize the two heteroreceptors. Compared with pH 7.4, the FRET signal was 3.87-fold higher at pH 6.2 within 1.5 h of incubation (Fig. 1f). HGF is the native ligand for c-Met [35]; therefore, its influence on the function of pH-CT was investigated. After stimulation with HGF, an increased FRET signal was still obtained under acidic conditions because the rigid steric hindrance caused by c-Met&TfR dimer blocked the binding epitope of c-Met to HGF (Fig. 1g, h). The result confirmed that the acidosis-induced *in situ* heterodimerization of receptors by pH-CT was invulnerable and irreversible. Confocal laser scanning microscopy verified the marked increase in red fluorescence on HepG-2 cells as _e_pH decreased from 7.4 to 6.2, with the strongest fluorescence intensity at pH 6.2 (Fig. 1i and Figs S6, S7). These results revealed that pH-CT could selectively dimerize heterogeneous receptors in response to acidic TME.

### Inhibition of cell migration and invasion by pH-CT

**Figure 2.**
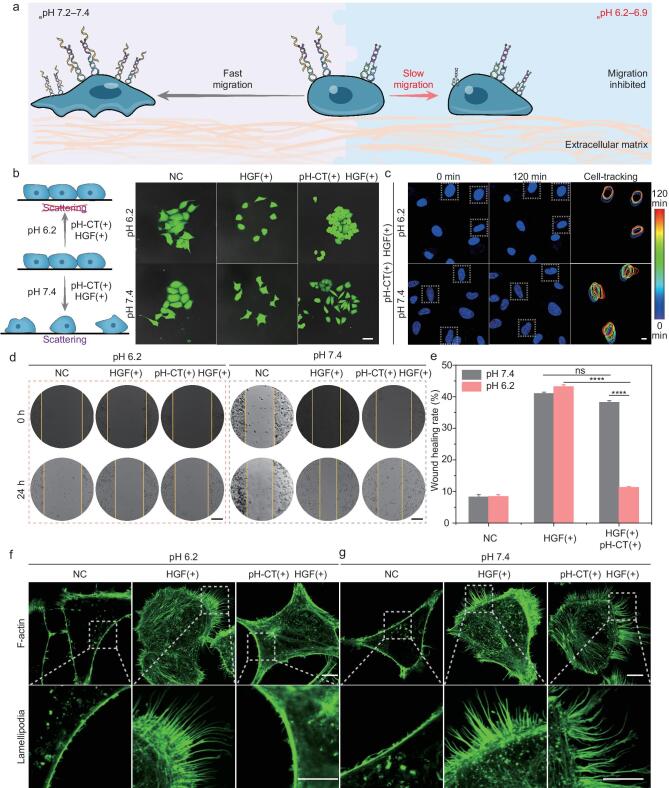
Precise inhibition of tumor cell migration. (a) Schematic illustration of pH-CT inhibition of cell migration. (b) Scattering of HepG-2 pretreated with pH-CT at different pH values after 24 h of HGF stimulation. Cells were stained with calcein-AM. Scale bar: 50 µm. (c) Migration tracking images of a single Hoechst-stained HepG-2. Scale bar: 10 µm. (d) Wound scratch results of cell mobility selectively abolished by pH-CT in HepG-2. Scale bar: 250 µm. (e) Statistical analysis of the relative acellular area of the wound scratch assay in (d) (*n* = 3). (f, g) Images of F-actin-rich lamellipodia on the HepG-2 surface after different treatments. Upper and lower scale bars: 10 µm. Statistical significance was calculated by two-tailed Student's *t*-test: ns, *P* > 0.05; ****, *P* < 0.0001.

Cell migration is the fundamental phenotype mediated by the c-Met/HGF signaling axis, and it was estimated by a cell scattering assay (Fig. 2a). HepG-2 cells were grown as colonies under normal and acidic conditions (NC group, Fig. 2b). Stimulation with HGF resulted in extensive dissociation of the colonies owing to enforced cell motility (HGF+ group). However, pH-CT suppressed this HGF effect at slightly acidic _e_pH compared with neutral conditions (pH-CT+, HGF+). This reduction in cell motility was comparable to that of cells treated with pH-CT alone. It should be noted that dissociation of cell colonies by HGF still occurred in target cells treated with a range of control probes (Figs S8, S9). A single-cell dynamic tracing assay was conducted via time-lapse fluorescence imaging. The cell migration trajectory suggested that cells treated with pH-CT under acidic conditions became nearly motionless (Fig. 2c and Fig. S10), whereas those treated at pH 7.4 maintained sustained migration trajectories (Fig. 2c and Fig. S11). We performed a scratch-wound healing test to clarify the inhibitory effect of pH-CT on HepG-2 cells. A significant delay in wound healing was observed in cells treated with pH-CT at pH 6.2 after 24 h of incubation (Fig. 2d). Quantitative analysis showed that the healing rate for pH-CT at pH 6.2 was 3.33-fold lower than that at pH 7.4 and 3.82-fold lower than that after HGF induction alone at pH 6.2 (Fig. 2e). Cell migration is known to be driven primarily by reorganization of the actin cytoskeleton [36], which provides propulsion for movement by polymerizing to generate lamellipodia [37]. Compared with HGF-treated or pH-CT-treated cells at pH 7.4, pH-CT-treated cells at pH 6.2 showed fewer F-actin–rich lamellipodia, resulting in significantly decreased motility (Fig. 2f, g). These results confirmed that pH-CT selectively inhibited cell migration by modulating endogenous receptor assembly according to abnormal _e_pH.

**Figure 3.**
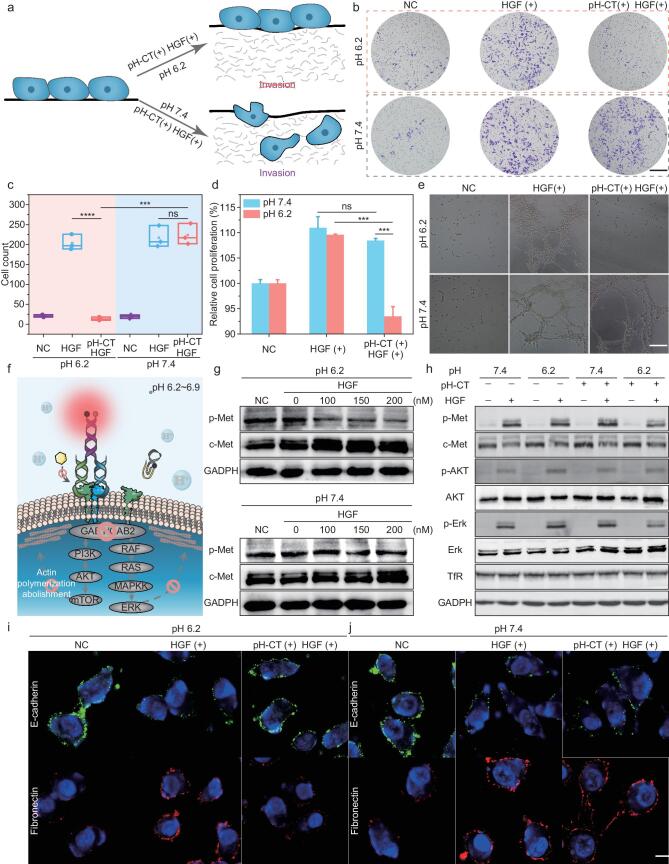
Selective inhibition of signaling events involving c-Met/HGF in HepG-2 cells treated with pH-CT. (a) Schematic illustration showing pH-CT inhibition of tumor invasion in a transwell assay. (b) Transwell assay of HepG-2 cells pretreated with pH-CT at different pH after HGF stimulation. Cells were stained with crystal violet. Scale bar: 200 µm. (c) Quantitative analysis of the number of invading cells in (b) (*n* = 3). (d) Viability of HepG-2 cells treated with pH-CT under different pH (*n* = 5). (e) Antiangiogenic effect of pH-CT in HUVEC tube-formation assay. Scale bar: 200 µm. (f) Schematic illustration of the working mechanisms of pH-CT. (g) The expression of p-Met in HGF-treated HepG-2 cells after treatment with different concentrations of pH-CT at neutral or acidic _e_pH. (h) Expression of downstream effectors of c-Met signal transduction in HepG-2 cells after treatment with pH-CT at different pH. Confocal fluorescence images of E-cadherin and fibronectin expression in HepG-2 cells after treatment with pH-CT at (i) pH 6.2 and (j) pH 7.4. Statistical significance was calculated by two-tailed Student's *t*-test: ns, *P* > 0.05; ***, *P* < 0.001; ****, *P* < 0.0001.

A transwell invasion assay was established using a Matrigel envelope. This test mimics the pattern of basement membrane cell invasion (Fig. 3a). The number of invading cells was reduced from 253 in cells treated with pH-CT at pH 7.4 to 10 in cells treated at acidic _e_pH (Fig. 3b, c). This implied that pH-CT could precisely attenuate cell motility and invasion based on mild acidosis. The effect of pH-CT on cell proliferation was then assessed. Treatment with HGF promoted 110.96% cell proliferation, whereas pH-CT at tumor-like pH abolished this and reduced the cell proliferation rate to 92.14% (Fig. 3d). In contrast, cells treated at normal _e_pH maintained an increased proliferation rate (108.73%). Finally, the antiangiogenic effect of pH-CT was assayed by tube-formation analysis utilizing HUVEC cells (Fig. S12). After co-incubation with pH-CT at pH 7.4, the cells formed a capillary-like network mediated by activation of the c-Met/HGF pathway. Nevertheless, pH-CT-treated cells grew apart under acidic conditions with little tube formation (Fig. 3e).

### Selective blockade of the HGF/c-Met signaling pathway by pH-CT

The capacity of pH-CT to antagonize c-Met-related downstream signaling was assessed by the expression of c-Met phosphorylation (p-Met). Western blotting (WB) showed that p-Met expression of cells at pH 6.2 exhibited a pH-CT dose-dependent reduction and was completely abolished at 200 nM (Fig. 3f, g). However, its content was hardly eliminated after treatment with the same concentration under neutral conditions. Such _e_pH-dependent antagonism would improve the specificity of targeted therapies compared with the traditional c-met inhibitor, such as foretinib (Fig. S13). Simultaneously, we investigated its potential to silence downstream signaling of c-Met, including trans-phosphorylation of protein kinase B (AKT) and extracellular signal-regulated kinase (Erk). An apparent decrease in p-AKT and p-Erk levels was observed when treated with pH-CT at acidic pH compared with p-AKT and p-Erk levels at pH 7.4 (Fig. 3h). Moreover, the control probes produced no obvious changes in p-Met or p-Erk levels, confirming the rational design of pH-CT within the environmental sense-allostery-reassembly procedure (Fig. S14). Epithelial-mesenchymal transition (EMT) is a fundamental biological process that transforms cells from an epithelial to a mesenchymal state, resulting in altered morphology, reduced cell-cell junctions and increased cell motility and migration [38]. Activation of the HGF/c-Met signaling pathway greatly facilitates the progression of EMT, with decreasing levels of E-cadherin [39] and increasing levels of fibronectin [40]. Therefore, the inhibitory effects of pH-CT on EMT were investigated based on the two EMT markers. Immunofluorescence imaging assay showed that pH-CT could effectively reverse the HGF-induced decrease in E-cadherin expression and increase in fibronectin expression under acidic conditions (Fig. 3i, j). This verified that pH-CT could selectively abolish the onset and progression of EMT based on TME cues.

### Inhibition of *in vivo* tumor metastasis by pH-CT

Encouraged by its *in vitro* performance, we evaluated the therapeutic applicability of pH-CT to metastatic tumors *in vivo*. First, its biostability was investigated under complex physiological conditions. The results indicated that pH-CT, even after allostery-reassembly, exhibited great structural stability within 10% fetal bovine serum (Fig. S15a), 40 μM glutathione (Fig. S15b), 200 μM cysteine (Fig. S15c), and 100 μM H_2_O_2_ (Fig. S15d) after 6 h treatments, since phosphorothioate skeletal decorations of oligonucleotides within pH-CT improve the stability (Table S1). The biosafety of pH-CT was estimated by measuring the hemolytic activities, body weight and organ toxicity of mice following pH-CT treatment. Unlike the apparent hemolysis induced by 1% Triton-X 100, erythrocytes retained their oval shape after incubation with pH-CT (hemolysis rate <4%), indicating its favorable hemocompatibility (Fig. S16). Similar to the PBS-treated group, no weight loss was noted in the pH-CT-treated mice (Fig. S17), and no obvious abnormalities were observed in organ coefficients (the ratio of organ weight to body weight) and biochemical indices (Figs S18–S20). Hematoxylin and eosin (H&E) staining of major organs confirmed no inflammation or lesions in the pH-CT-treated group (Fig. S21). These results suggested the favorable biocompatibility of pH-CT in mice.

We used the HepG-2 tumor-bearing xenograft nude mouse model to investigate the ability of pH-CT to target tumors within circulation, utilizing an aptamer and i-motif co-inactivated nanoassembly, npH-nCT, as a negative control probe. Whole-body fluorescence imaging indicated that pH-CT enabled efficient activation at the tumor site, achieving maximum contrast at 1 h with a tumor-to-normal tissue ratio (T/N) of 3.38 (Fig. S22). Conversely, lower contrast was achieved after injection of the control probe (T/N 1.35, Fig. S23). These results confirm that pH-CT can achieve precise *in vivo* tumor imaging by aptamer-driven membrane-tethering and low pH-triggered turn-on FRET signal.

**Figure 4.**
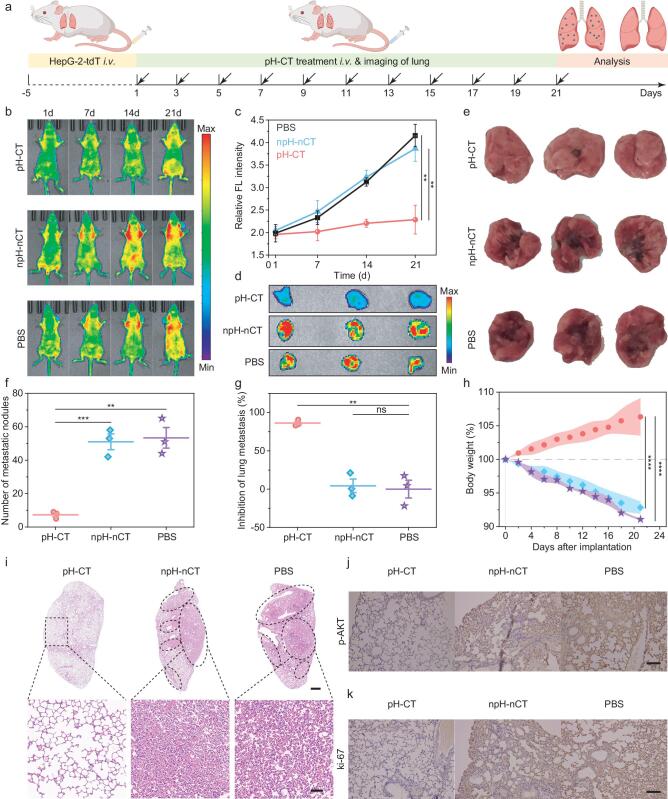
*In vivo* antimetastatic therapeutic efficacy of pH-CT. (a) Schematic illustration of the treatment timeline for early hepatocarcinoma pulmonary metastasis in mice. (b) Fluorescence imaging of lung metastases in mice after different treatments at 1, 7, 14 and 21 days. (c) Quantitation of tdTomato fluorescence in mice after different treatments at defined time points (*n* = 3). (d) *Ex vivo* fluorescence imaging of lung tissue harvested from mice after treatments. (e) Photographs of lungs excised from HepG-2-tdT pulmonary metastasis mice after different treatments. (f) Statistical analysis of pulmonary metastatic foci from different treatments (*n* = 3). (g) Suppression of lung metastasis by different treatments (*n* = 3). (h) Body weight fluctuations during treatments (*n* = 3). (i) H&E staining of lung tissues from treated mice. Upper scale bar: 500 µm. Lower scale bar: 50 µm. (j and k) Immunohistochemistry of (j) p-AKT and (k) ki-67 levels in lung tissues. Scale bar: 100 µm. Statistical significance was calculated by two-tailed Student's *t*-test: **, *P* < 0.01; ***, *P* < 0.001; ****, *P* < 0.0001.

Tumor metastasis involves the intravasation of cancer cells into adjacent blood or lymphatic vessels [41] and the subsequent escape of tumor cells from the lumen of these vessels into the distant tissue parenchyma, where they form small nodules and eventually progress to a macroscopic tumor [42]. The lung, as the first filter of systemic circulation, is the most vulnerable organ in the process of extrahepatic metastasis [43]. Therefore, a mouse model of early hepatocarcinoma pulmonary metastasis was established by intravenously administering HepG-2-tdT cells into BALB/c-nu mice, after which we injected pH-CT or npH-nCT intravenously every 2 d to assess its anti-invasion and antimetastatic potential by recording the signal of *in vivo* tdTomato fluorescent protein from lungs (Fig. 4a). The tdTomato signal increased slightly by 1.05-fold in the pH-CT-treated group on day 21 after treatment (Fig. 4b, c). However, the npH-nCT- and PBS-treated groups showed obvious fluorescence enhancements (1.9-fold and 2.12-fold increase, respectively), indicating numerous metastatic lesions in the lungs. *Ex vivo* fluorescence photography of lung tissue harvested after treatments indicated that the pH-CT-treated group displayed minimal fluorescence compared with the control groups, confirming the pronounced therapeutic effect of pH-CT (Fig. 4d). By counting the number of metastatic lesions visible on the surface of lung tissues, the pH-CT-treated group presented the fewest metastatic tumor nodules (7 ± 2) relative to the npH-nCT- and PBS-treated groups by 13.73% and 13.12%, respectively (Fig. 4e, f). pH-CT treatment resulted in 86.25% suppression of lung metastasis, much higher than that of npH-nCT treatment (Fig. 4g). The weight loss caused by lung metastasis was also reversed by pH-CT (Fig. 4h). The inhibition of lung metastasis by pH-CT was validated by histological analysis. H&E staining showed a uniform reticular morphology of pulmonary alveoli in the pH-CT group, whereas lung tissues from npH-nCT- and PBS-treated groups both showed severe lung metastases with massive neutrophil infiltration (Fig. 4i).

Immunohistochemistry showed lower p-AKT in lung tissues from the pH-CT-treated group compared with the npH-nCT- and PBS-treated groups (Fig. 4j), suggesting that pH-CT impeded tumor metastasis via inhibition of the c-Met/AKT signaling pathway. Ki-67 is a key marker of aberrant tumor proliferation and acts as a reliable indicator of response to intervention [44]. The lungs of pH-CT-treated mice showed a decrease in Ki-67 compared with the other two groups, demonstrating the inhibitory effect of pH-CT on pulmonary metastasis (Fig. 4k). These results manifested that pH-CT inhibited pulmonary metastasis from hepatocarcinoma by disrupting c-Met-mediated tumor migration and invasion.

## CONCLUSION AND PERSPECTIVE

We leveraged the aberrant TME of metastatic lesions to design an _e_pH-driven DNA nanodevice with tailored inhibition of tumor metastasis *in vivo*. Existing DNA nanoinhibitors, such as spl3c [27] and DBL-4C5 [28] aptamers, mainly adopt occupancy-driven pharmacology to interrupt receptor function. However, with this mechanism of action, the therapeutic effects are outweighed by unfavorable side effects or false signals caused by the ‘always on’ design principle [45]. Interest has been growing in surmounting this obstacle by developing stimuli-activatable DNA nanoassemblies that specifically perform gene silencing [46], protein degradation [47], and immune checkpoint blockade [48] in response to tumor-exclusive cues or external instructions. Indeed, strategies that incorporate control factors into precision medicine are highly desirable. Currently, advances in functional nucleic acids (FNAs) have given DNA nanotechnology the ability to respond to various external stimuli, such as light [49], enzymes [50], and small molecules [51], and has provided compelling tools for nongenetic regulation of receptor functions in a user-delimited fashion. Although these DNA nanoassemblies offer opportunities to modulate receptor-mediated cellular behavior, it is rare to use oncogenic stimulus-responsive DNA nanodevices to antagonize hyperactivated signaling axes with tailored *in vivo* antimetastatic functions. In addition, present approaches are mainly based on static or permanent DNA patterns, which are not suitable for precise and tunable blockade of cellular processes involved in metastasis. The field of receptor-mediated antimetastatic therapeutics is still in its infancy, and *in vivo* suppression of metastasis with improved tumor selectivity has not yet been achieved.

We considered these challenges and integrated i-motif-based allosteric molecule protectors with nontherapeutic aptamers to create an orthogonal acidosis-mediated antimetastatic strategy applicable to *in vivo* interception of hyperactive signaling receptors, in particular c-Met. Remarkably, compared with most other studies that have focused on modulating cellular behavior, our research offers the first example of triggerable DNA assembly in precise *in vivo* antimetastatic therapeutics by clustering heterogeneous receptors with high-order tumor selectivity. The pH-CT developed in our study harnessed the programmable and predictable properties of DNA nanotechnology, as characterized by a number of achievements. First, with pH-CT, we have established a paradigm for prototypical DNA nanodevices for efficient and precise antimetastatic therapy. Second, the dynamic running machinery of pH-CT overcomes the drawback of poor manipulation efficiency and promises noninvasive and more effective antimetastatic therapy via *in situ* DNA self-reassembly on oncogenic receptors. Third, pH-CT eradicates unfavorable toxic effects owing to its _e_pH-reliant inhibitory activity. Fourth, the _e_pH-induced heterodimerization of membrane receptors is irreversible, exerting a sustained inhibitory activity on the hyperactive c-Met/HGF axis signaling pathway. Such a long-lasting effect enables the dose and frequency of administration to be reduced, leading to a further reduction of side effects.

pH-CT provides an additional TME-controlled dimension to the suppression of metastasis, supplementing existing radiological and chemotherapeutic antimetastatic treatments, and it is expected to provide new opportunities for the development of aptamer-based drugs and vaccines. This unparalleled potential allows pH-CT to be modified with special synthetic-biological functions, such as acidosis-mediated activation of T cells. In addition, pH-CT could reprogram native c-Met to perceive two levels of messages: TME assessment and interaction with nearby heterogeneous receptors, which is congruent with the operating mechanism of native ion-channel proteins, such as Na^+^/K^+^-ATPase. In this regard, pH-CT could provide a band-pass _e_pH filter for biomedical engineering, allowing a greater curative effect or more precise identification of metastasis in tumorigenesis and progression.

The future objective is to develop other TME (e.g. hypoxia [52] and high ATP levels [53])-driven immunogenic cell death through a stimulus-triggered DNA feedback network to entirely reform *in vivo* antimetastatic strategies. Unlike previous efforts that have focused on modulating cell behavior, our strategy uses rationally designed FNAs to disrupt metastasis-related cellular processes in a nongenetic pathway, which is amenable to off-the-shelf applications for *in situ* antagonism of metastases. This customized approach provided a plug-and-play implementation for various types of hyperactive receptor signaling. pH-CT could chemically functionalize diverse oncogenic receptors as TME-responsive devitalization outputs by simply replacing Apt-Met and Apt-TfR with aptamers that target proteins of interest, in turn simplifying the engineering of receptor blockade and targeted protein degradation.

Despite this, the clinical translation of pH-CT may be hindered by efficient *in vivo* delivery and renal excretion due to its nucleic acid nature [54]. Chemical functionalization of the oligonucleotides, such as DNA chirality adjustment [55,56], nanoparticle loading [57], and artificial nucleoside incorporations [58], etc., will considerably improve their *in vivo* circulating half-life while increasing structural stability, offering practical options for prospective clinical implementation. In addition, FNAs-based medicines typically need to be delivered accurately to specific tissues or organs to meet various disease treatment demands. Unfortunately, the specificity and even the function of existing aptamers are compromised in sophisticated physiological systems, as the working conditions in living environments are far from the screening conditions [59]. Therefore, further research should be aimed at optimizing the design of current FNAs to suit practical applications, as well as adopting different screening circumstances according to different clinical requirements to generate FNAs with more efficient performance. Besides, the simple diffusion of components in oligonucleotide-based drugs may cross-link the unwanted tissues [19]. To overcome this challenge and minimize the off-target effect, it's better to develop an all-in-one nanoassembly by integrating all recognition elements on a stable scaffold.

In summary, we exploited FNAs and DNA dynamic feedback to develop a TME-actuated DNA nanodevice for precise *in vivo* antimetastatic therapy. This modular strategy affords flexible and versatile recoupling of metastatic-tumor-generated microenvironmental inputs with tailored receptor-perturbation outputs, as well as sensitive _e_pH responsiveness in precise antimetastatic therapeutics. The *in vivo* assessment substantiated the remarkable therapeutic benefits of the DNA nanodevice in mitigating the incidence of pulmonary metastasis with 86.25% inhibition in an early hepatocarcinoma-lung-metastasis mouse model. Our findings elucidate the function of the _e_pH-activated DNA nanodevice in inhibiting metastasis-related cellular processes and may open up new avenues for the development of effective antimetastatic treatments in the future.

## METHODS

The experimental details are given in the Supplementary data. All animal experiments in this study were performed in accordance with ethical protocols/guidelines (SYXK (Yu) 2018–0004) approved by the Laboratory Animal Center of Henan Province, China.

## Supplementary Material

nwae471_Supplementary_Data
