# Supplementary material for: An epH-driven DNA nanodevice for impeding metastasis in vivo by selectively blocking cell signaling
Source: Natl Sci Rev. 2025 Jan 7;12(3):nwae471. doi: 10.1093/nsr/nwae471 (PMC11879464; doi:10.1093/nsr/nwae471)
Supplement: nwae471_Supplementary_Data [file nwae471_Supplementary_Data.pdf]

Supplementary Data

An $_e$pH-driven DNA Nanodevice for Impeding Metastasis *In Vivo* by Selectively Blocking Cell Signaling

Kun Yuan[1,†], Hong-Min Meng[1,†], Hongzhi Sun[1], Lingbo Qu[1], Zhaohui Li[1,2,*] & Weihong Tan[3,4,*]

[1]College of Chemistry, Institute of Analytical Chemistry for Life Science, Zhengzhou University, Zhengzhou 450001, China

[2]The First Affiliated Hospital of Zhengzhou University, Zhengzhou University, Zhengzhou 450052, China

[3]Zhejiang Cancer Hospital, Hangzhou Institute of Medicine (HIM), Chinese Academy of Sciences, Hangzhou, Zhejiang 310022, China

[4]Institute of Molecular Medicine (IMM), Renji Hospital, Shanghai Jiao Tong University School of Medicine, and College of Chemistry and Chemical Engineering, Shanghai Jiao Tong University, Shanghai 200240, China

[†]These authors contributed equally: Kun Yuan, Hong-Min Meng.

*Corresponding Authors: Prof. Zhaohui Li and Prof. Weihong Tan

*Correspondence to: zhaohui.li@zzu.edu.cn and tan@him.cas.cn

Content

1. Experimental Procedures S4

1.1 Materials and Reagents S4

1.2 Cell culture S4

1.3 Animals S5

1.4 Experimental section S5

1.4.1 Construction of pH-CT S5

1.4.2 Characterization of pH-CT S5

1.4.3 Fluorescence evaluation in test tubes S6

1.4.4 Flow cytometric test S6

1.4.5 Confocal microscopy imaging S6

1.4.6 Western blot assay S7

1.4.7 Cell scattering analysis S7

1.4.8 Single-cell dynamic tracing assay S7

1.4.9 Wound-healing assay S8

1.4.10 Cytoskeleton analysis S8

1.4.11 Cell invasion analysis S9

1.4.12 Cell proliferation assay S9

1.4.13 Antiangiogenic assay S9

1.4.14 Biosafety exploration S10

1.4.15 *In vivo* tumor imaging S10

1.4.16 *In vivo* antimetastatic therapy S10

1.4.17 H&E staining analysis S11

1.4.18 Immunohistochemical staining analysis S11

2. Supplementary Figures S12

Figure S1. FRET behavior of pH-CT. S12

Figure S2. FRET spectrum of pH-CT. S13

Figure S3. FRET efficiency of pH-CT. S14

Figure S4. Receptor expression on HepG-2 cells. S15

Figure S5. Potential of pH-CT to target receptors on cellular surface. S16
Figure S6. Acidosis-induced heterogeneous protein dimerization by pH-CT. ...S17
Figure S7. Quantitative analysis of fluorescence signal on cell membrane. S18
Figure S8. Schematic representation of different control probes. S19
Figure S9. Cell scattering analysis of different control probes. S20
Figure S10. Single-cell dynamic tracing assay of activated pH-CT...................S21
Figure S11. Single-cell dynamic tracing assay of inert pH-CT..........................S22
Figure S12. Receptor expression on HUVEC cells. S23
Figure S13. Inhibitory efficacy of a traditional c-met inhibitor on p-Met level in HGF-treated HepG-2 cells under different pH conditions. S24
Figure S14. Difference between pH-CT and control probes in signaling pathway blockage. S25
Figure S15. Stability evaluation of pH-RE. S26
Figure S16. Hemolysis assay of pH-CT...S27
Figure S17. Body weight analysis after pH-CT treatment................................S28
Figure S18. Organ coefficients analysis after pH-CT treatment. S29
Figure S19. Hematological parameters analysis after pH-CT treatment. S30
Figure S20. Biochemistry parameters analysis after pH-CT treatment. S32
Figure S21. H&E analysis after pH-CT treatment. S33
Figure S22. Accurate *in vivo* tumor imaging by pH-CT. S34
Figure S23. Difference between pH-CT and control probe in T/N ratio. S35
3. Supplementary Table S36
Table S1. DNA sequences used in this work..S36

1. Experimental Procedures

1.1 Materials and Reagents

All oligonucleotide sequences (detailed in Supplementary Table 1) were synthesized and HPLC-purified by Sangon Biotech Co., Ltd. (Shanghai, China). Dulbecco's modified Eagle's medium (DMEM), fetal bovine serum (FBS), and Dulbecco's phosphate-buffered saline (PBS) were all purchased from GIBCO Invitrogen Corp. and Synergy Brands, Inc. (SYBR) Gold, Calcein-AM, and Hoechst-33342 were obtained from Sigma-Aldrich Co., Ltd. (St. Louis, MO). FITC-labeled phalloidin, crystal violet, trypsin-EDTA, penicillin & streptomycin, RIPA lysis buffer, phosphatase inhibitor, PMSF, and antibody diluent were purchased from Beyotime Institute of Biotechnology (China). Matrix gel and 6.5 mm Transwell® with 8.0 µm Pore Polycarbonate Membrane Insert were purchased from Corning Inc. (USA). Cell Counting Kit-8 was purchased from Sangon Biotech Co., Ltd. (Shanghai, China). Recombinant Human HGF (Hepatocyte Growth Factor, CJ72) was purchased from Novoprotein (China). Antibodies for phospho-MET (Tyr1234/1235, #3077), Met (D1C2, #8198), phospho-Akt (Ser473, #4060), Akt (#9272), phospho-Erk1/2 (Thr202/Tyr204, #4370), Erk1/2 (#9102), TfR (D7G9X, #13113) and GADPH (D16H11, #5174) were obtained from Cell Signaling Technology (USA).

1.2 Cell culture

The human hepatoblastoma cell lines HepG2 and HepG2-tdT and the human umbilical vein endothelial cell line HUVEC were purchased from the Cell Resource Center, Peking Union Medical College. HepG2, HepG2-tdT, and HUVEC cells were cultured in DMEM medium supplemented with 10% fetal bovine serum (FBS), 1% penicillin and 100 U/mL streptomycin at 37 ℃ in a humidified 5% CO_2 atmosphere.

1.3 Animals

The 8-week-old female BALB/c-nu mice were acquired from SPF Biotechnology Co., Ltd. All animal experiments were performed in accordance with ethical protocols/guidelines (no. SYXK (Yu) 2018-0004) approved by the Laboratory Animal Center of Henan Province (China).

1.4 Experimental section

1.4.1 Construction of pH-CT

The nanodevice consists of two subcomponents, Apt-Met/i-motif-M and Apt-TfR/i-motif-T. Each subcomponent was organized by annealing the capture strand (Apt-c-Met or Apt-TfR) with its corresponding i-motif. Specifically, for Apt-Met/i-motif-M, Apt-Met and i-motif-M were mixed at a molar ratio of 1:1.2 in 5 mM Mg^{2+} PBS (pH 7.4) to obtain the optimal signal-to-background ratio and then heated at 95 ℃ for 5 min, followed by cooling to room temperature overnight. The Apt-TfR/i-motif-T subcomponent was prepared by mixing Apt-TfR and i-motif-T in a molar ratio of 1:1.8 and then following the same procedure. Equal quantities of constructed Apt-Met/i-motif-M and Apt-TfR/i-motif-T together form pH-CT.

1.4.2 Characterization of pH-CT

To evaluate the sense-allostery-reassembly potential of pH-CT, 9% PAGE was conducted. pH-CT was pipetted into PBS (5 mM Mg^{2+}) of different pH (pH 7.4 and pH 6.2) to give a final concentration of 1 μM and then incubated at 37 °C for 1 h. Afterwards, the above samples mixed with 2μL 6×loading buffer and 2μL SYBR Gold were loaded on 9% PAGE. Electrophoresis was then conducted in 1× TBE buffer at 80 V for 1.5 h in an ice bath. Finally, the collected gel was analyzed by using the Molecular Imager Gel Doc XR system (Bio-Rad Laboratories, Hercules, CA).

1.4.3 Fluorescence evaluation in test tubes

To validate its sense-allostery-reassembly performance, pH-CT was diffused to a final concentration of 100 nM in a series of pH PBS (pH 6.0~7.4) and then incubated at 37℃ for 1h. All fluorescence data were monitored on a fluorescence spectrophotometer (Hitachi F-7100, Tokyo, Japan). Emission spectra, ranging from 655 nm to 750 nm, were recorded at an excitation wavelength of 633 nm with a slit width of 10 nm. All measurements were repeated at least three times. Original fluorescence data were assayed via Origin-2018.

1.4.4 Flow cytometric test

To estimate its ability to induce heterodimerization between c-Met and TfR receptors on cell membrane, 200nM pH-CT were incubated with 2×10^5 HepG-2 cells in 200 μL different DMEM (pH 6.2, and 7.4) for a certain time (0.5h, 1.0h, 1.5h, and 2.0h) at 37 ℃ in the dark. After washing with DPBS three times, the cells were assayed with a flow cytometer (Gallios, Beckman Coulter, Brea, CA).

1.4.5 Confocal microscopy imaging

HepG-2 cells were seeded at a density of 5×10^3 cells on 15 mm glass-bottomed dishes and incubated with 200 nM pH-CT in FBS-free DMEM medium with a pH gradient (pH 6.2-7.4) at 37℃ for 1.5 hours in the dark. Following triplicate washing with DPBS, cell photographs were captured on a Leica TCS SP8 confocal laser scanning fluorescence microscope (Wetzlar, Germany) with a 100× oil immersion objective. Cy3 fluorescence signal was collected in the yellow channel with 550-nm excitation, Cy5-FRET fluorescence signal was collected in the red channel with 550-nm excitation, and Hoechst-33342 fluorescence was collected in the blue channel with 405-nm excitation. For cell scattering analysis, the cells were stained with Calcein-AM for 20 min at 37℃. Calcein-AM fluorescence was obtained in the green channel with 488-nm excitation. For the single-cell dynamic tracing assay, cells were

marked with Hoechst-33342 for 15 min at 37 ℃.

1.4.6 Western blot assay

Following incubation with 200 nM pH-CT at pH 6.2 in FBS-free DMEM and induction with 200 ng/mL HGF, cells were lysed in RIPA lysis buffer with the addition of protease inhibitors and phosphatase inhibitors. The cell lysates were centrifuged at 12000 rpm for 15 min to discard cellular debris. Combined with 5× loading buffer, the protein lysates were boiled at 100 ℃ for 15min, and the samples were further assayed by electrophoresis on a 10% SDS-polyacrylamide gel. After separation, the samples were transferred onto a 0.45μm PVDF membrane (Millipore, USA) for 130 min using a wet transfer system. Blocked with 5% non-fat milk solutions for 60 min, the membranes were immersed in personal primary antibodies overnight at 4 ℃ and in matching secondary antibodies for 60 min at room temperature. All antibodies were diluted at 1:1500 ~ 1:3000. After three washes with TBST, the marked membranes were then reacted with ECL substrate solution for 5 min. Chemiluminescence results were acquired using ChemiDoc-XRS+ (Bio-Rad, USA).

1.4.7 Cell scattering analysis

HepG-2 cells (~ 200 cells per well) were plated on 15 mm glass-bottomed dishes at 37 ℃ under 5% CO_2 for 3 days. After colony growth, cells were incubated with 200 nM pH-CT for 1.5 h under different pH DMEM (pH 7.4 and 6.2), washed three times with DPBS, inoculated with 200 ng/mL HGF, and then incubated for a further 24 h. The morphology of the treated cells was then observed by confocal microscopy.

1.4.8 Single-cell dynamic tracing assay

HepG-2 cells (~ 100 cells per well) were plated on 15 mm glass-bottomed dishes at 37 ℃ under 5% CO_2 for 12h. Afterwards, the cells were pre-stained with Hoechst

for 15 min. The prestained cells were incubated with 200 nM pH-CT for 1.5 h in different pH DMEM (pH 7.4 and 6.2), washed three times with DPBS, inoculated with 200 ng/mL HGF, and then monitored using a real-time online incubation and imaging system (Wetzlar, Germany).

1.4.9 Wound-healing assay

First, HepG-2 cells (5×10^6 cells per well) were seeded in a 24-well plate and cultured overnight. When the cells reached 80~90% confluence, each cell monolayer was uniformly scratched by a sterile 10 μL pipette tip and then washed three times with DPBS to remove the floating cells. Then, the cells were incubated with 200 nM pH-CT for 1.5 h under different pH DMEM (pH 7.4 and 6.2), washed three times with DPBS, inoculated with 200 ng/mL HGF, and incubated for a further 24 h. Cell migration area was acquired at 0 and 24 h via a digital inverted microscope (AMG evos). Cells without any treatment were set as a control group.

1.4.10 Cytoskeleton analysis

HepG-2 cells (~ 1×10^3 cells per well) were plated on 15 mm glass-bottomed dishes at 37 ℃ under 5% CO_2 for 24h. The cells were incubated with 200 nM pH-CT for 1.5 h under different pH DMEM (pH 7.4 and 6.2), washed three times with DPBS, inoculated with 200 ng/mL HGF, and incubated for a further 24 h. Next, the cells were fixed with 1% paraformaldehyde for 20 min at 25 ℃. After washing three times with DPBS, cells were further permeabilized with 0.5% (v/v) Triton X-100 in DPBS for 10 min at 25 ℃. After that, the treated cells were stained with FITC-labeled phalloidin for 0.5h. After three washes with DPBS, fluorescence images of the cytoskeleton were captured using a Leica TCS SP8 confocal laser scanning fluorescence microscope (Wetzlar, Germany) with a 100× oil immersion objective.

1.4.11 Cell invasion analysis

Briefly, ~2×10^4 HepG-2 cells were seeded overnight on the matrix gel-covered upper chamber (8 μm pore size) of a 24-well transwell plate for attachment. The attached cells were incubated with 200 nM pH-CT for 1.5 h in DMEM of different pH (pH 7.4 and 6.2), washed three times with DPBS, inoculated with 200 ng/mL HGF, and then incubated for a further 24 h. The medium in the upper chamber was then replaced with 200 ng/mL HGF-containing FBS-free DMEM, and the lower chamber was filled with 500 μL DMEM containing 20% FBS to stimulate cell invasion. The chamber was maintained at 37 ℃ for a further 24 h. After three washes with DPBS, cells in the upper chamber were fixed with 1% paraformaldehyde for 20 min at 25 ℃ and then stained with 2% crystal violet for 20 min at 25 ℃. The invading cells were visualized via a digital inverted microscope (AMG evos).

1.4.12 Cell proliferation assay

CCK-8 reagent was used to assess the effect of pH-CT on cell viability. Typically, HepG-2 cells (~1×10^3 cells per well) were plated into a 96-well plate at 37 ℃ under 5% CO_2 for 24h. Subsequently, the attached cells were incubated with 200 nM pH-CT for 1.5 h in DMEM of different pH (pH 7.4 and 6.2), washed three times with DPBS, inoculated with 200 ng/mL HGF, and incubated for a further 48 h. Then the treated cells were mixed with 10 μL CCK-8 kit for 4 h at 37 ℃ with 5% CO_2. Finally, the optical absorption of formazan at 490 nm was monitored by a microplate reader (Spark, Männedorf, Switzerland). Each well was repeated six times.

1.4.13 Antiangiogenic assay

Around 5×10^4 HUVEC cells were seeded into the matrix gel-covered 24-well plate for attachment. The attached cells were incubated with 200 nM pH-CT for 1.5 h in DMEM of different pH (pH 7.4 and 6.2), washed three times with DPBS, inoculated with 200 ng/mL HGF, and incubated for a further 6 h. After incubation, the

formatted tube was observed using a digital inverted microscope (AMG evos).

1.4.14 Biosafety exploration

BALB/c-nu mice without any treatment were injected intravenously with PBS and 2.0 nmol pH-CT to assess biosafety. Blood samples were collected at the end of the treatments for routine blood tests and analysis of biochemical indices. 10 μL whole blood were used for the routine blood test. 200μL of collected plasma were used for evaluation of blood biochemical indices. Harvested major organs were fixed in 4% paraformaldehyde and sliced to a thickness of 4 μm for H&E staining to investigate the histopathologic toxicity to tissues.

1.4.15 *In vivo* tumor imaging

Each BALB/c-nu mouse was subcutaneously injected with 1×10^7 HepG-2 cells in 50 μL DPBS in the right shoulders to obtain a subcutaneous tumor model. The tumor was allowed to grow for 10 to 15 days until reaching 0.8~1.2 cm in diameter. Then mice were intravenously administrated 200 μL of DPBS buffer containing 2.0 nmol of pH-CT and 5 nmol of random oligonucleotides. Time-lapse fluorescence imaging of the treated mice was acquired on an IVIS Lumina II *in vivo* imaging system (Caliper Life Science, Waltham, MA). A 550 nm bandpass filter was chosen as the excitation filter, and a 680 nm band-pass filter was used as the Cy5-FRET emission filter.

1.4.16 *In vivo* antimetastatic therapy

In vivo antimetastatic therapy was investigated in lung metastatic hepatoma models. Around 5×10^6 of HepG-2-tdT cells were intravenously administrated into BALB/c-nu mice to generate the pulmonary metastasis model. Five days later, 9 mice were equally and randomly divided into three groups (pH-CT, npH-nCT, and PBS). Then 200 μL of DPBS buffer containing 2.0 nmol of pH-CT (or npH-nCT, or PBS

only) were injected into mice (every two days) via the tail vein. At the appointed time (0, 7, 14, and 21 days) after administration, mice were assayed for tdTomato fluorescence from lung metastatic tumors by an IVIS Lumina II *in vivo* imaging system (Caliper Life Science, Waltham, MA). Afterwords, lungs harvested from mice in each group were imaged.

1.4.17 H&E staining analysis

After 21 days of treatment, all mice were sacrificed. Harvested lungs were fixed in 4% paraformaldehyde for 24 h. After dehydration through a series of alcohols (75% alcohol for 4h, 85% alcohol for 2h, 90% alcohol for 2h, 95% alcohol for 1h, and anhydrous ethanol for 0.5h, 1:1 (V: V) benzene and alcohol mixture for 5 min and xylene for 10 min), the lungs were embedded in paraffin using an embedding machine. Afterwards, the wax blocks were sliced into 5μm-thick sections. After deparaffinization and rehydration, lung slices were stained with Hematoxylin solution for 5 min and rinsed again with ddH_2O. After that, the slices were treated with Hematoxylin Differentiation solution, Hematoxylin Scott Tap Bluing, and Eosin dye, respectively. Sealed with neutral gum, the slices were visualized via a digital inverted microscope (AMG evos).

1.4.18 Immunohistochemical staining analysis

In brief, deparaffinized lung sections were immersed in CH_3COONa antigen retrieval solution (pH 6.0) and then blocked with 3% BSA for 0.5h. Lung sections were hatched sequentially with p-AKT or Ki-67 primary antibody overnight at 4 ℃ and with HRP-labeled secondary antibody for another 1h. Then the lung sections were stained with 3,3 N-Diaminobenzidine Tetrahydrochloride and counterstained with the hematoxylin staining solution for a few minutes. After dehydration, the sections were covered with BioMount medium for imaging.

2. Supplementary Figures

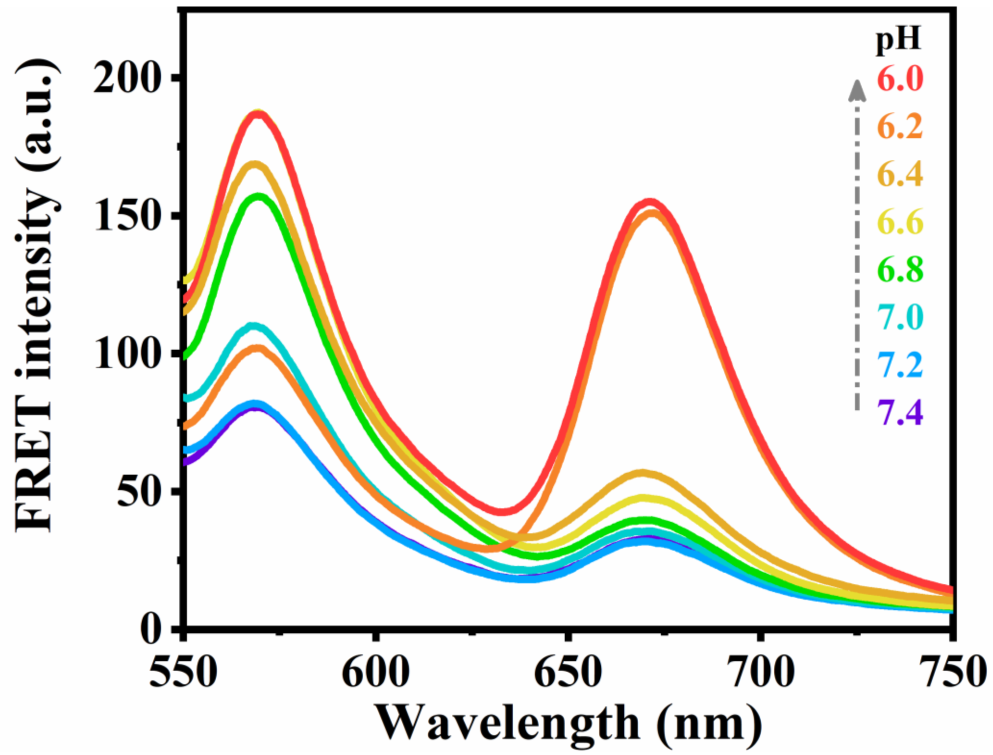

Figure S1. FRET behavior of pH-CT. FRET fluorescence behavior of pH-CT at different pH values.

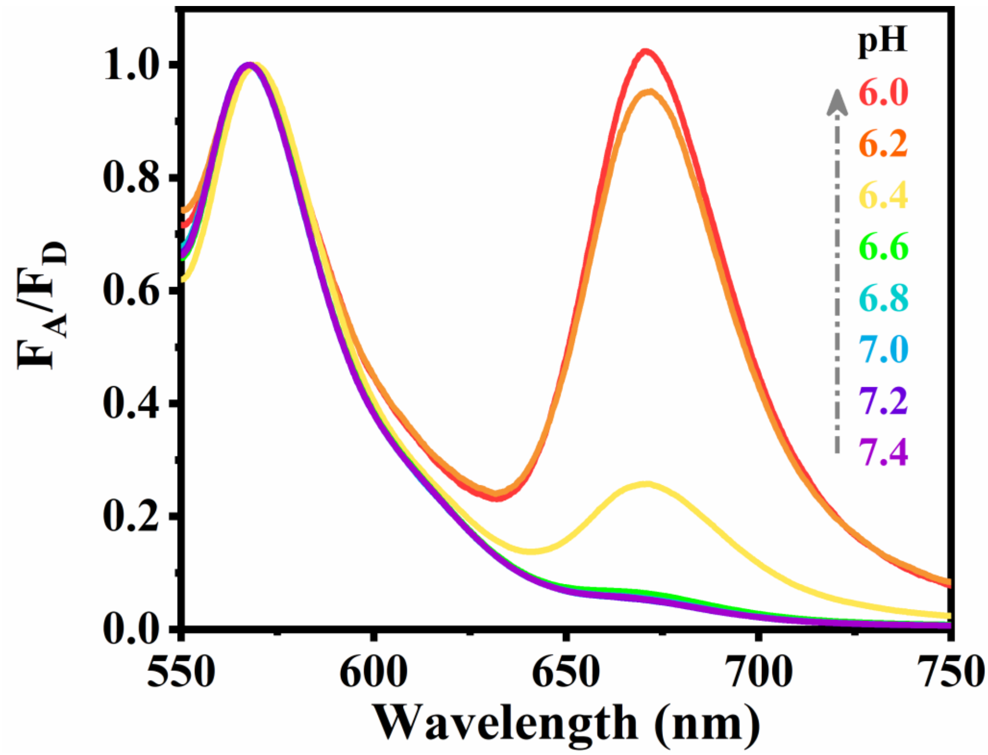

Figure S2. FRET spectrum of pH-CT. Fluorescence spectra of the auxiliary of pH-CT responding to a series of pH values.

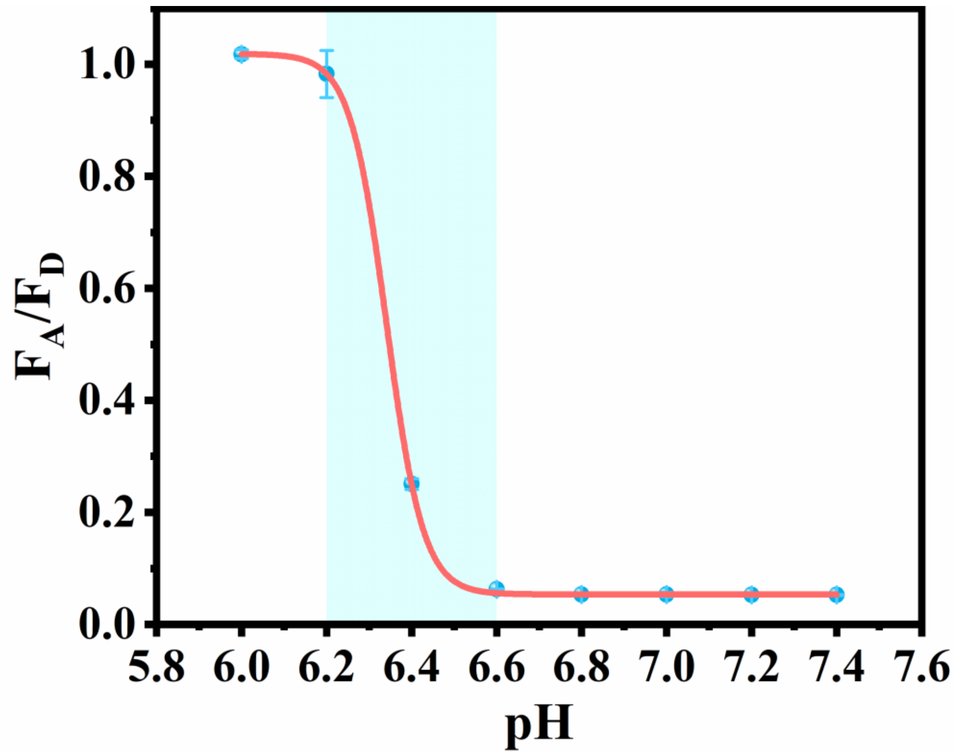

Figure S3. FRET efficiency of pH-CT. Plot of the fluorescence signal ratio of Cy5 to Cy3 (F_A/F_D) of pH-CT versus pH values. Error bars represent the standard deviations of three parallel tests.

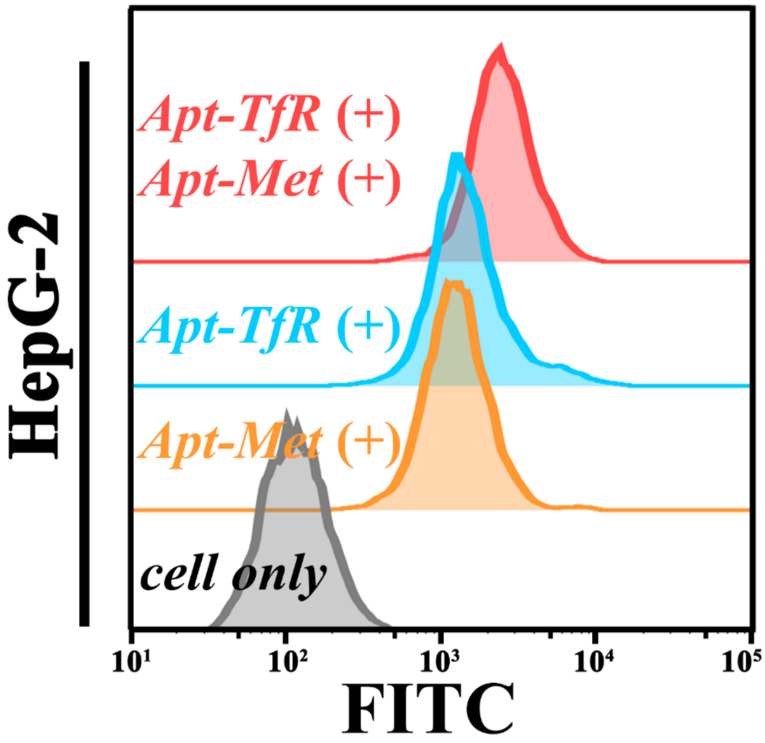

Figure S4. Receptor expression on HepG-2 cells. Verification of the expression of c-Met and TfR receptors on HepG-2 cells by flow cytometry.

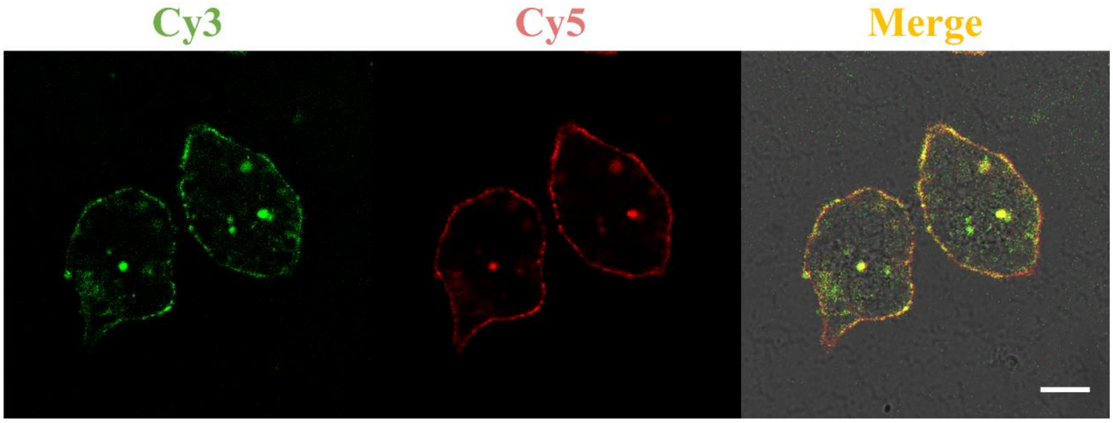

Figure S5. Potential of pH-CT to target receptors on cellular surface. Confocal fluorescence images of HepG-2 cells treated with pH-CT without BHQ-2-labelling ("fluorescence always-on" Cy3-Apt-Met/i-motif-M and Cy5-Apt-TfR/i-motif-T). Scale bars: 10 μm.

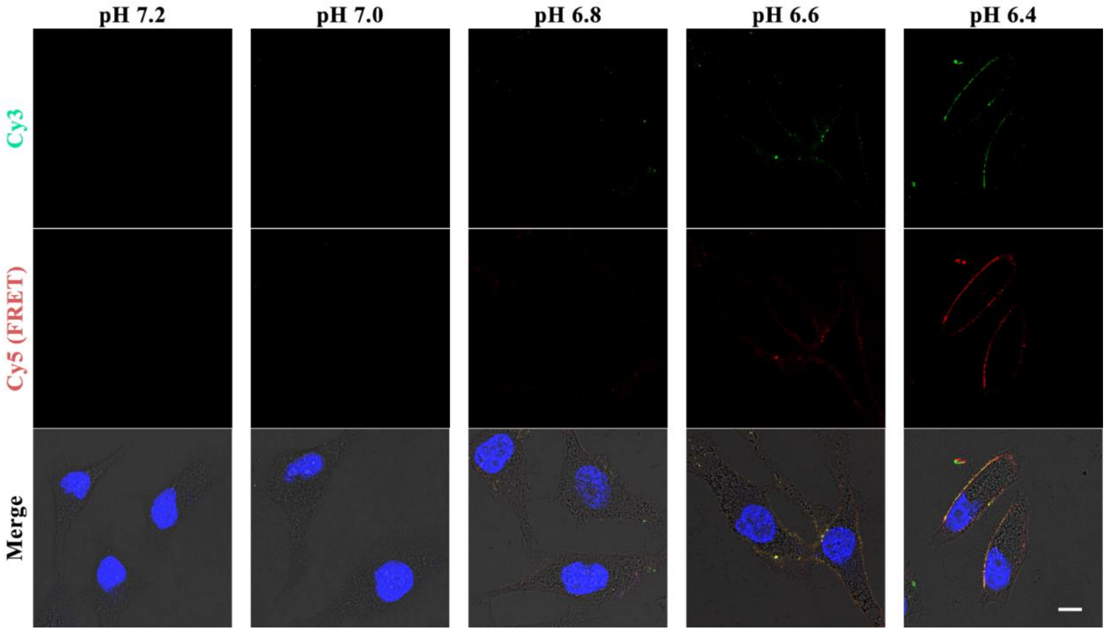

Figure S6. Acidosis-induced heterogeneous protein dimerization by pH-CT. Confocal fluorescence images of HepG-2 cells treated with pH-CT under different pH. Scale bars: 10 μm.

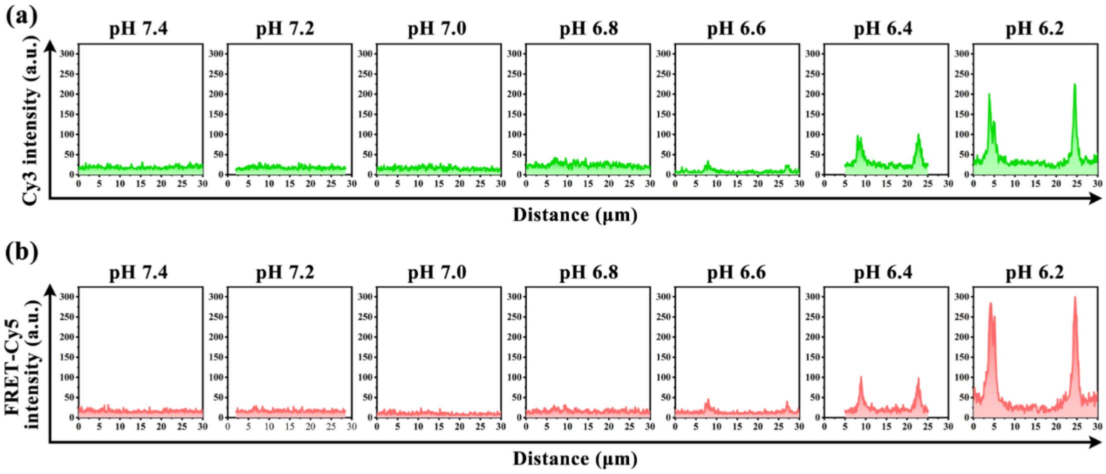

Figure S7. Quantitative analysis of fluorescence signal on cell membrane. Fluorescence intensities of (a) Cy3- and (b) Cy5-FRET as a function of the distance along the white arrows in Figure 1i and Figure S5.

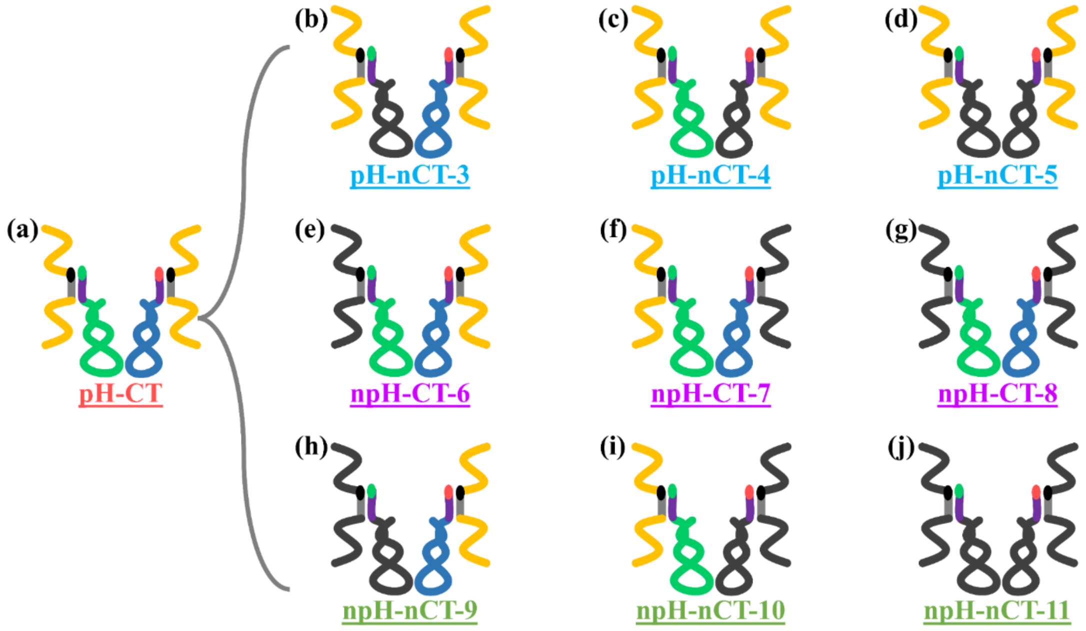

Figure S8. Schematic representation of different control probes. Black domains represent random sequence. As the first control, the key region of Apt-Met or Apt-TfR or both was substituted with random sequences to yield pH-nCT-3, 4 and 5. As the second control, the i-motif of either one or both subcomponents of pH-CT was substituted with pH-immobile sequences to give npH-CT-6, 7 and 8. As the third control, npH-nCT-9, 10, and 11, either one or both subcomponents were mutated.

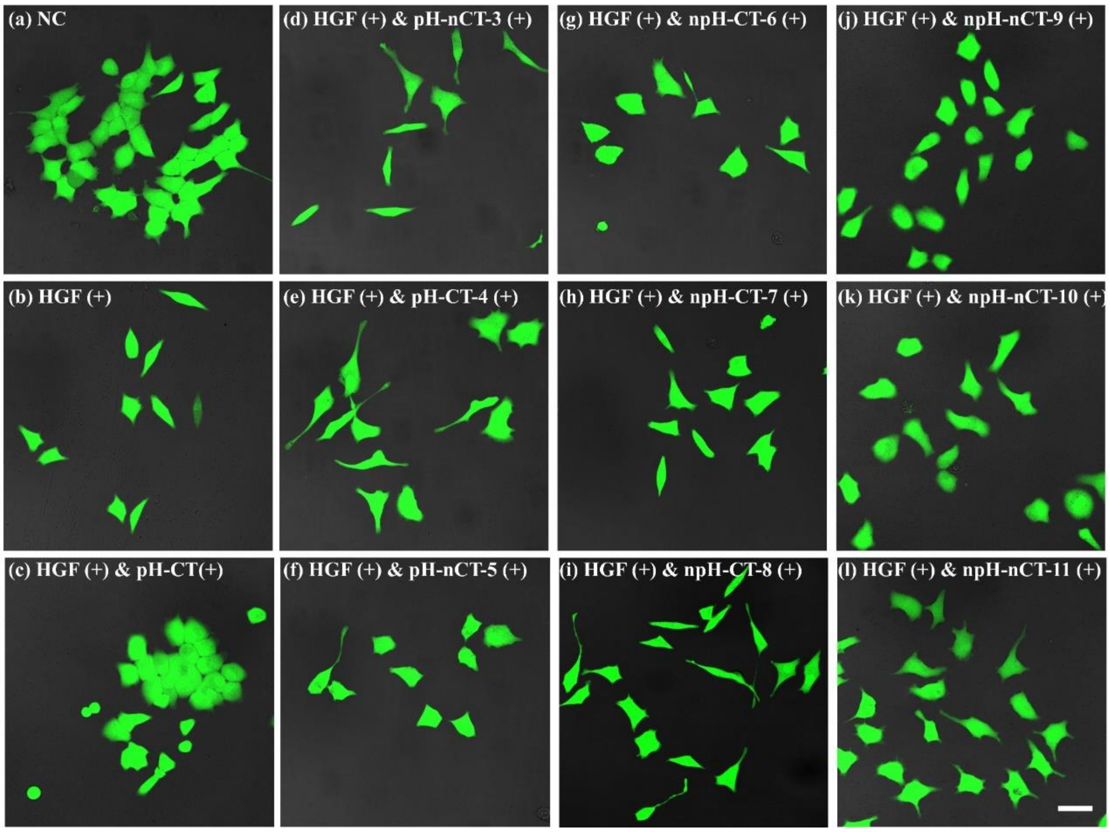

Figure S9. Cell scattering analysis of different control probes. Cell scattering analysis of HepG-2 cells pretreated with a series of control probes in DMEM (pH 6.2) containing HGF after 24 h. Cells were stained with Calcein-AM. (a) NC, (b) HGF (+), (c) HGF (+) and pH-CT (+), (d) HGF (+) and pH-nCT-3 (+), (e) HGF (+) and pH-nCT-4 (+), (f) HGF (+) and pH-nCT-5 (+), (g) HGF (+) and npH-CT-6 (+), (h) HGF (+) and npH-CT-7 (+), (i) HGF (+) and npH-CT-8 (+), (j) HGF (+) and npH-nCT-9 (+), (k) HGF (+) and npH-nCT-10 (+), and (l) HGF (+) and npH-nCT-11 (+). Scale bar: 50 μm.

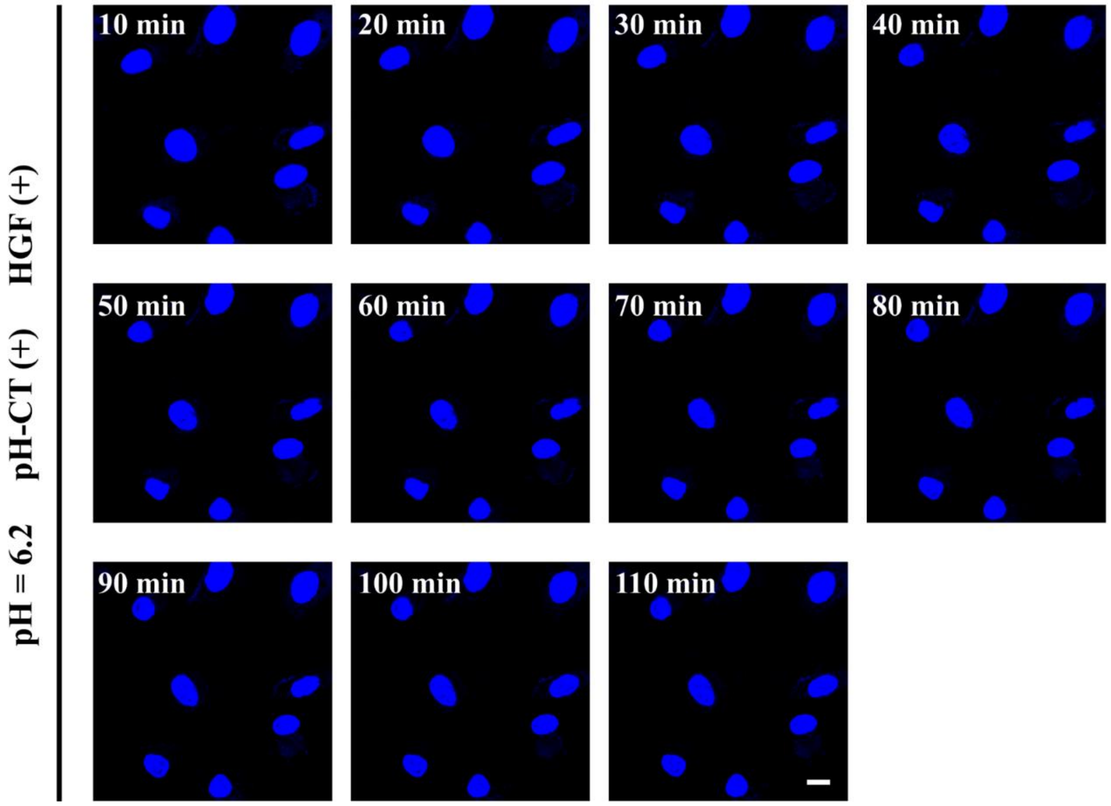

Figure S10. Single-cell dynamic tracing assay of activated pH-CT. Fluorescence images of migration tracking of one single Hoechst-stained HepG-2 cell that was pretreated with pH-CT in pH 6.2 DMEM containing HGF. Scale bar: 10 µm.

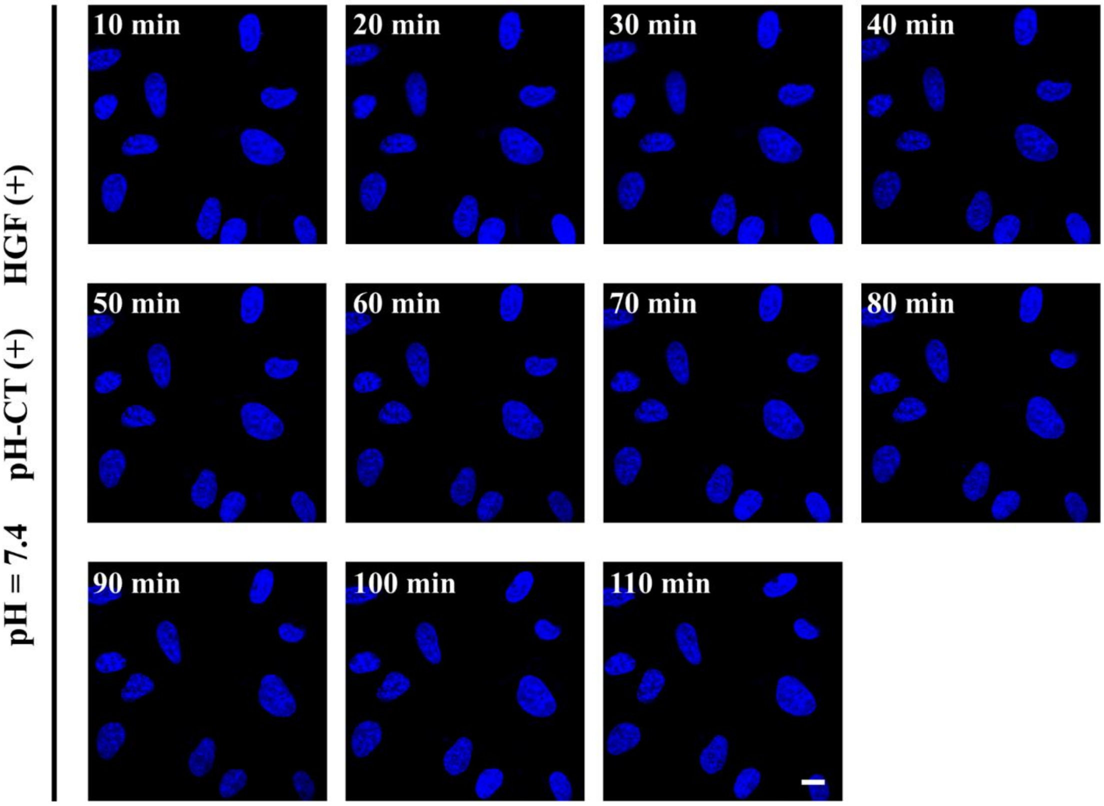

Figure S11. Single-cell dynamic tracing assay of inert pH-CT. Fluorescence images of migration tracking of one single Hoechst-stained HepG-2 cell that was pretreated with pH-CT in pH 7.4 DMEM containing HGF. Scale bar: 10 μm.

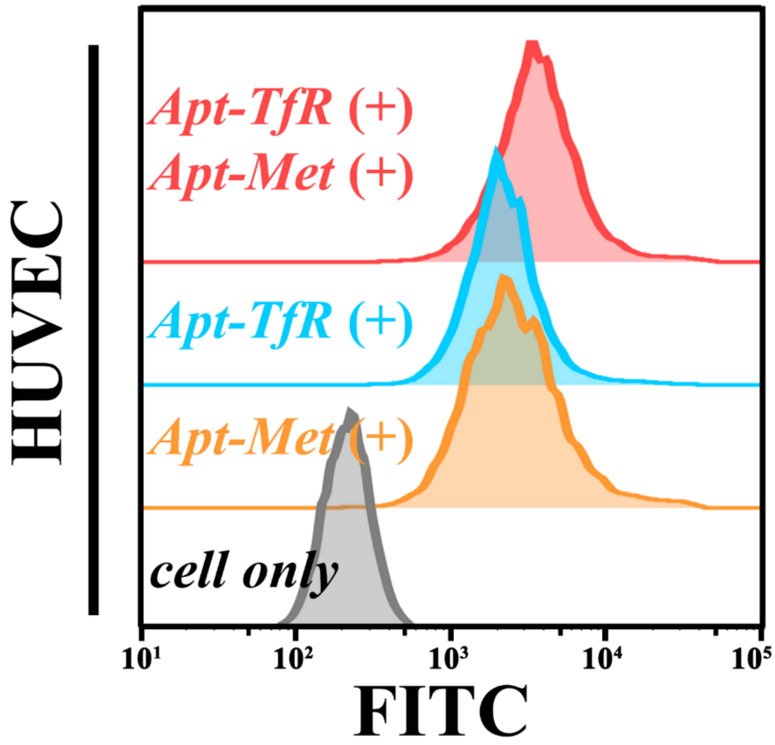

Figure S12. Receptor expression on HUVEC cells. Verification of the expression of c-Met and TfR receptors on HUVEC cells by flow cytometry.

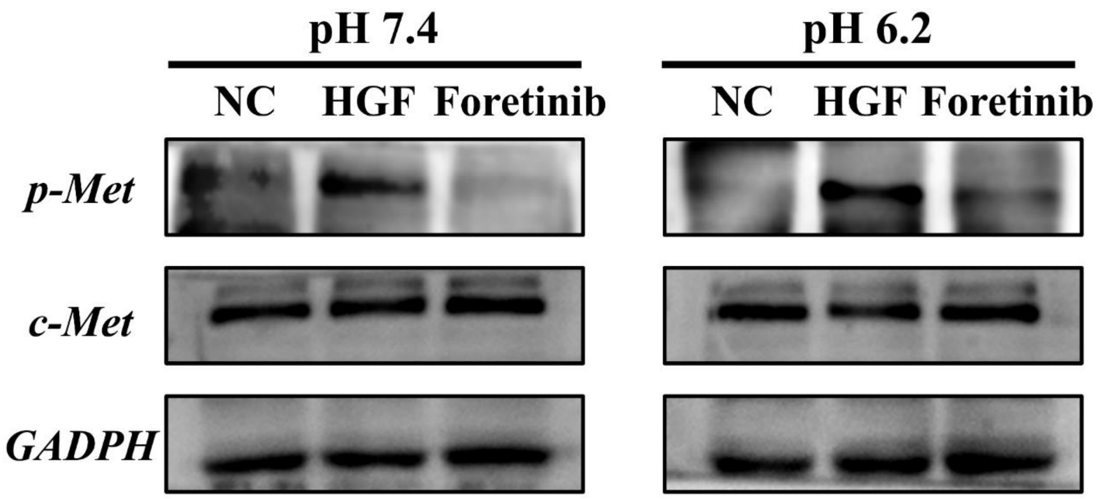

Figure S13. Inhibitory efficacy of a traditional c-met inhibitor on p-Met level in HGF-treated HepG-2 cells under different pH conditions. Inhibitory efficacy of traditional c-met inhibitor, foretinib (500 nM), on p-Met level in HGF-treated HepG-2 cells at pH 7.4 or pH 6.2.

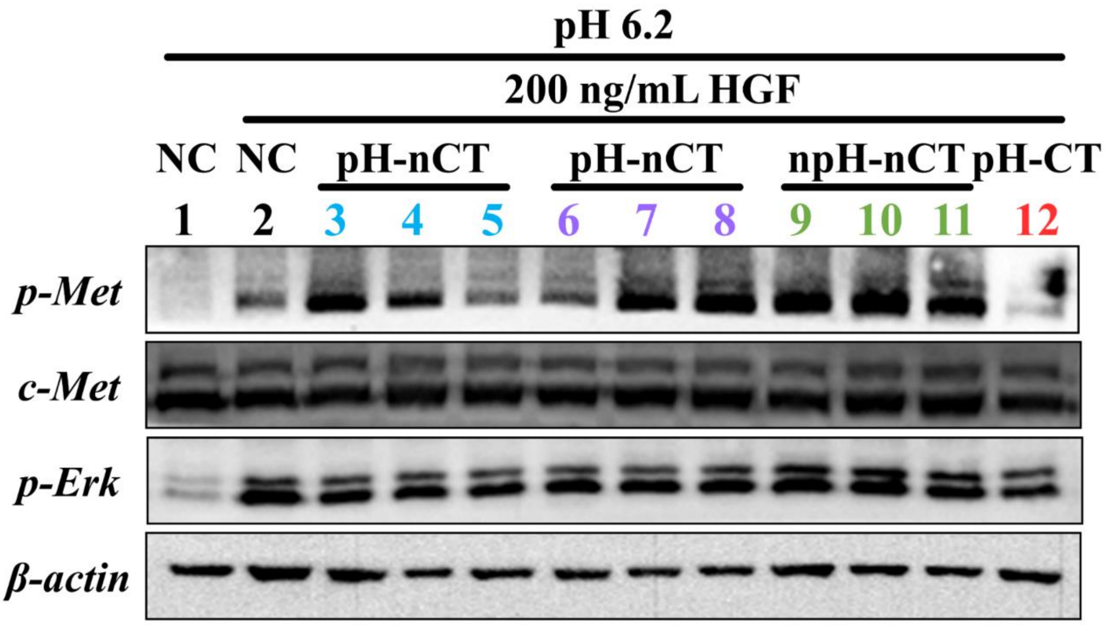

Figure S14. Difference between pH-CT and control probes in signaling pathway blockage. Inhibitory efficiency of pH-CT and different negative control probes on p-Met and p-Erk expression in HGF-treated HepG-2 cells at pH 6.2.

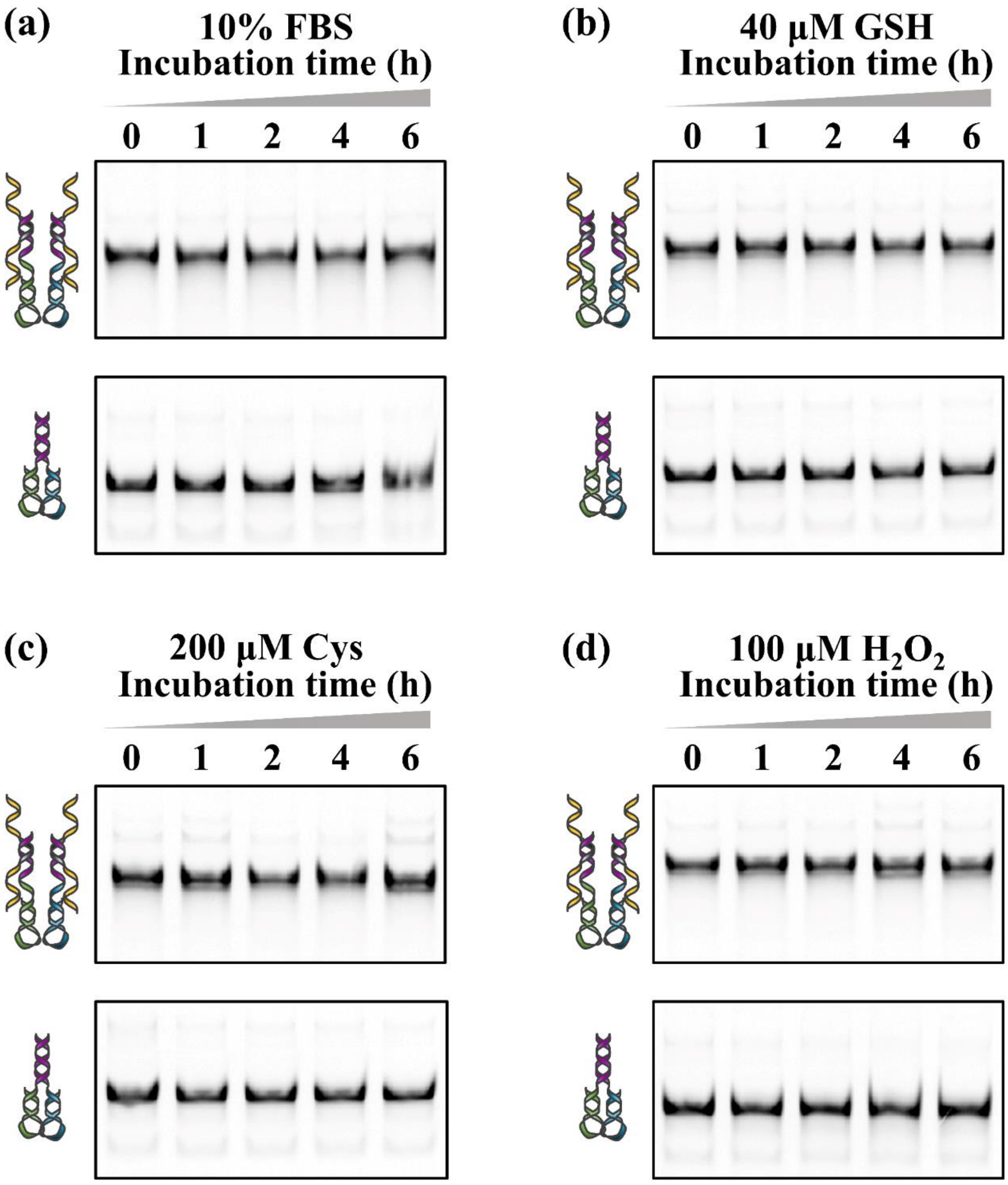

Figure S15. Stability evaluation of pH-RE. (a) After incubation in 10% fetal bovine serum (FBS). (b) After incubation in 40 μM glutathione (GSH). (c) After incubation in 200 μM cysteine (Cys). (d) After incubation in 100 μM H_2O_2.

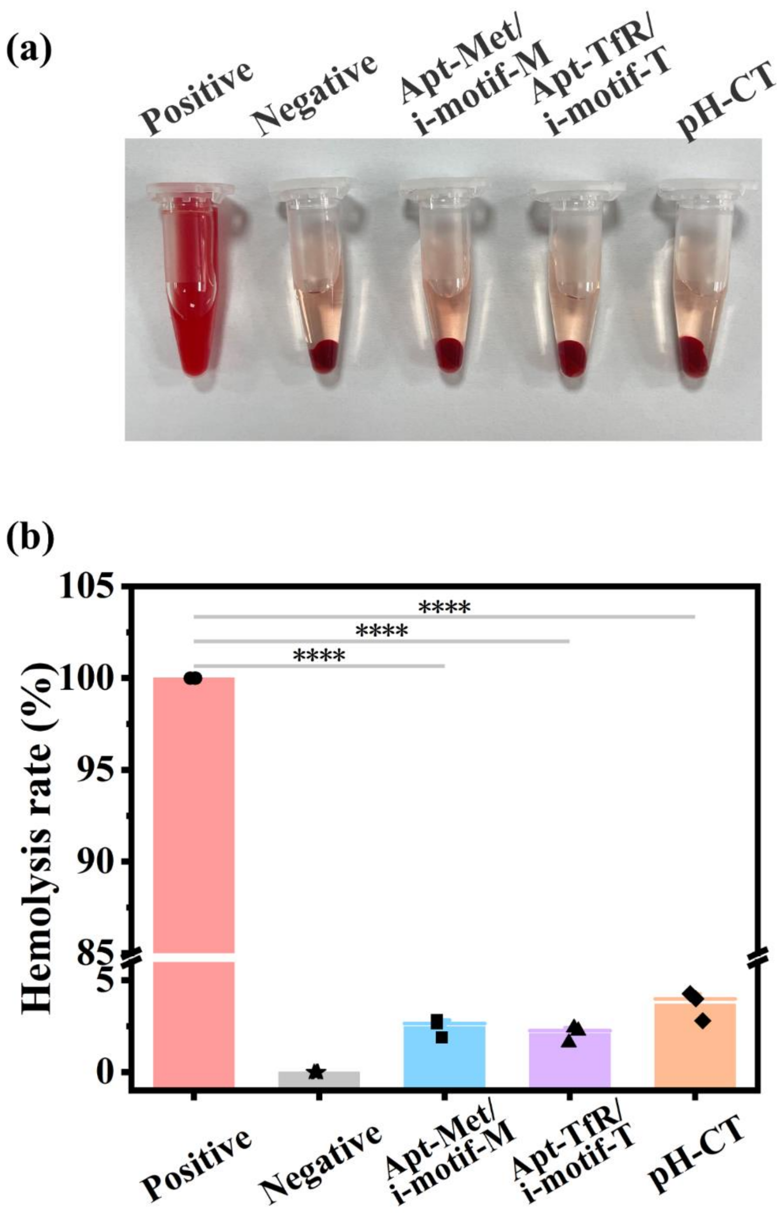

Figure S16. Hemolysis assay of pH-CT. (a) The photo images and (b) hemolysis ratio of red blood cells cocultured with pH-CT (n=3). Red blood cells incubated with 1 % Triton X-100 were used as a positive control. A hemolysis rate of <4% was regarded as no obvious hemolysis. Statistical significance was calculated by two-tailed Student's t-test: ****, P < 0.0001.

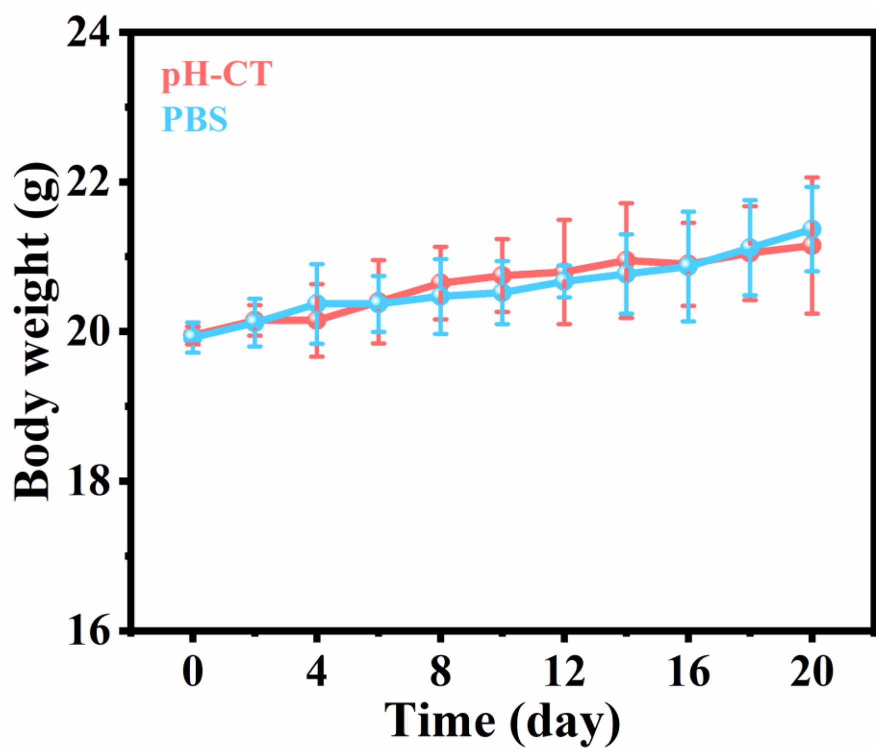

Figure S17. Body weight analysis after pH-CT treatment. Body weight change in mice treated by pH-CT or PBS (n=3).

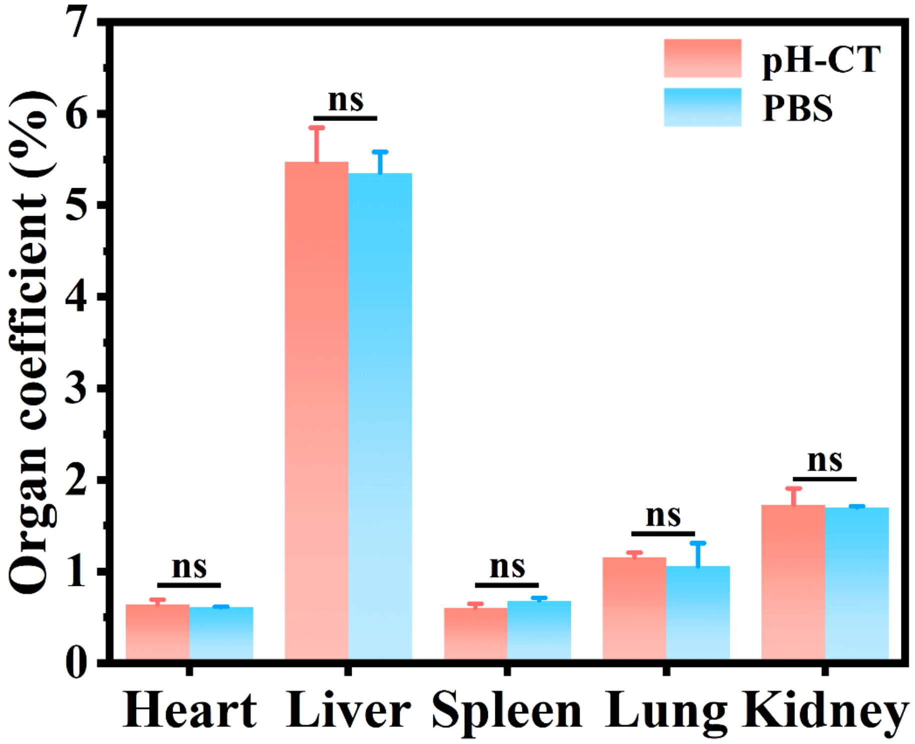

Figure S18. Organ coefficients analysis after pH-CT treatment. Organ coefficients of mice on the 21st day after treatment (n=3). Statistical significance was calculated by two-tailed Student's t-test: ns, $P > 0.05$.

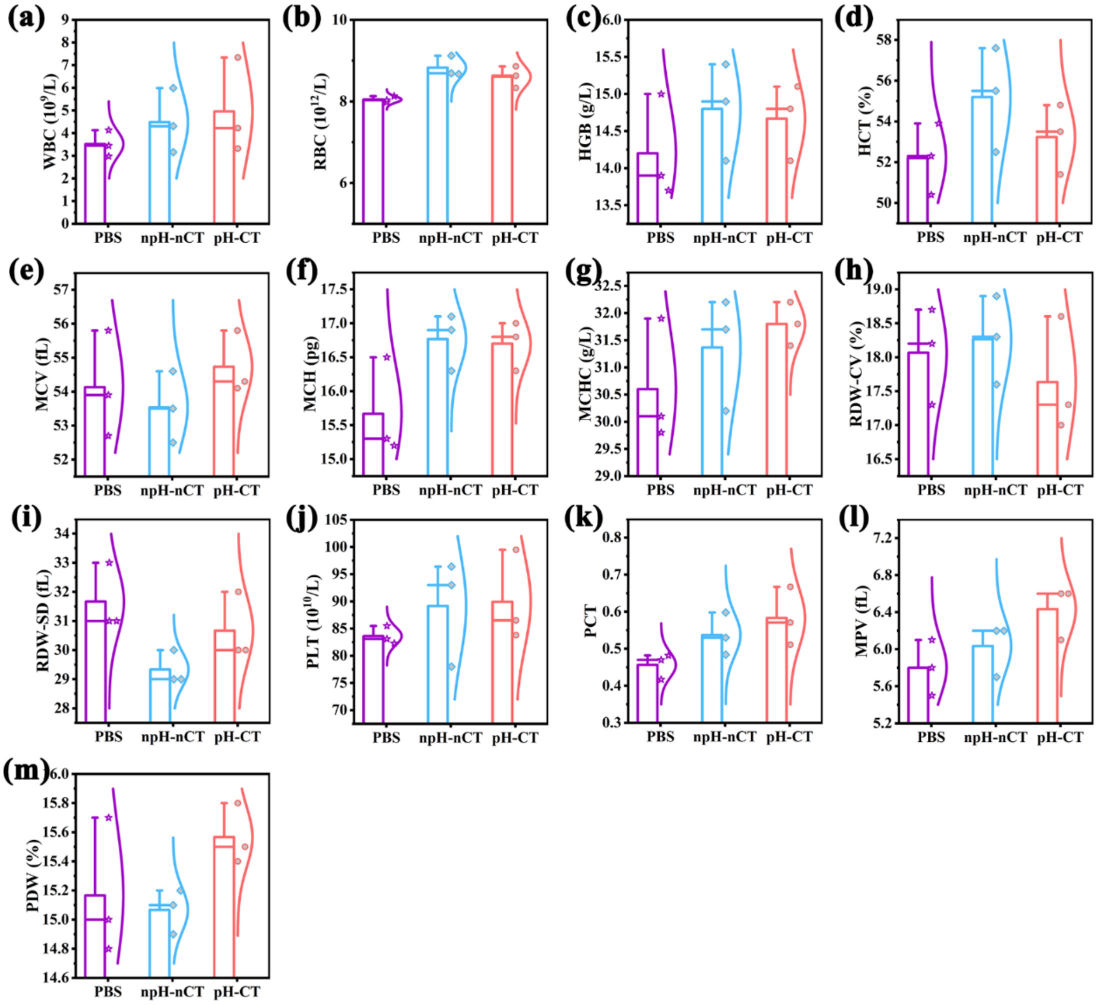

Figure S19. Hematological parameters analysis after pH-CT treatment. Hematological parameters in blood from mice 21 days post-treatment (n=3). (a) WBC: white blood cells. (b) RBC: red blood cells. (c) HGB: hemoglobin. (d) HCT: hematocrit. (e) MCV: mean corpuscular volume. (f) MCH: mean corpuscular hemoglobin. (g) MCHC: mean corpuscular hemoglobin concentration. (h) RDW-CV: red blood cell distribution width. (i) RDW-SD: red blood cell distribution width standard deviation. (j) PLT: platelet. (k) PCT: plateletcrit. (l) MPV: mean platelet volume. (m) PDW: platelet distribution width.

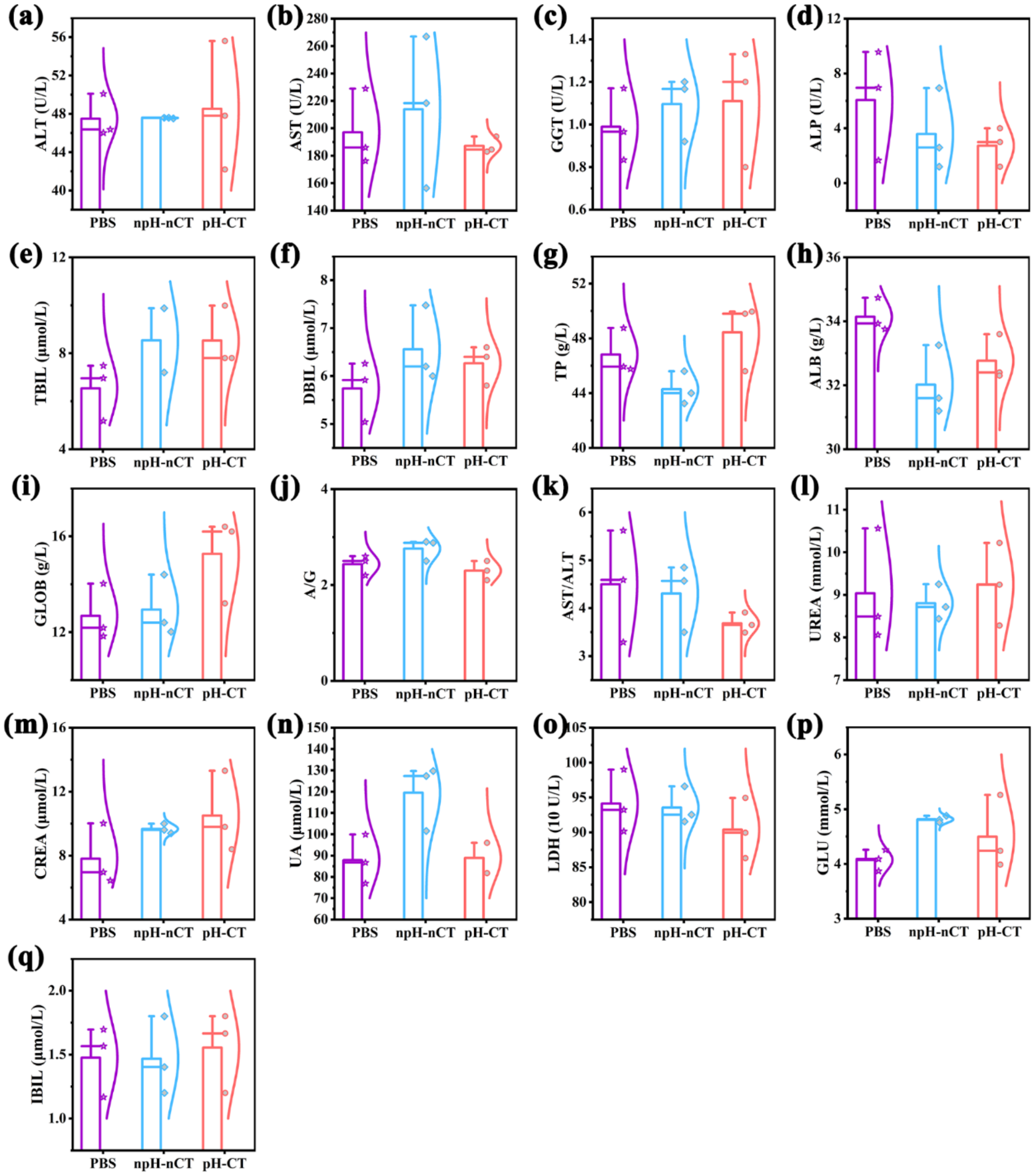
(a)
ALT (U/L)
(b)
AST (U/L)
(c)
GGT (U/L)
(d)
ALP (U/L)
(e)
TBIL (μmol/L)
(f)
DBIL (μmol/L)
(g)
TP (g/L)
(h)
ALB (g/L)
(i)
GLOB (g/L)
(j)
A/G
(k)
AST/ALT
(l)
UREA (mmol/L)
(m)
CREA (μmol/L)
(n)
UA (μmol/L)
(o)
LDH (10 U/L)
(p)
GLU (mmol/L)
(q)
IBIL (μmol/L)
PBS
npH-nCT
pH-CT

Figure S20. Biochemistry parameters analysis after pH-CT treatment. Biochemistry parameters in blood from mice 21 days post-treatment (n=3). (a) ALT: glutamic-pyruvic transaminase. (b) AST: aspartate aminotransferase. (c) GGT: Gamma Glutamyl Transpeptidase. (d) ALP: alkaline phosphatase. (e) TBIL: total bilirubin. (f) DBIL: direct bilirubin. (g) TP: total protein. (h) ALB: albumen. (i) GLOB: globulin. (j) A/G: albumen/globulin. (k) AST/ALT: aspartate aminotransferase/glutamic-pyruvic transaminase. (l) UREA: urea. (m) CREA: creatinine. (n) UA: uric acid. (o) LDH: lactate dehydrogenase. (p) GLU: glucose. (q) IBIL: Indirect Bilirubin.

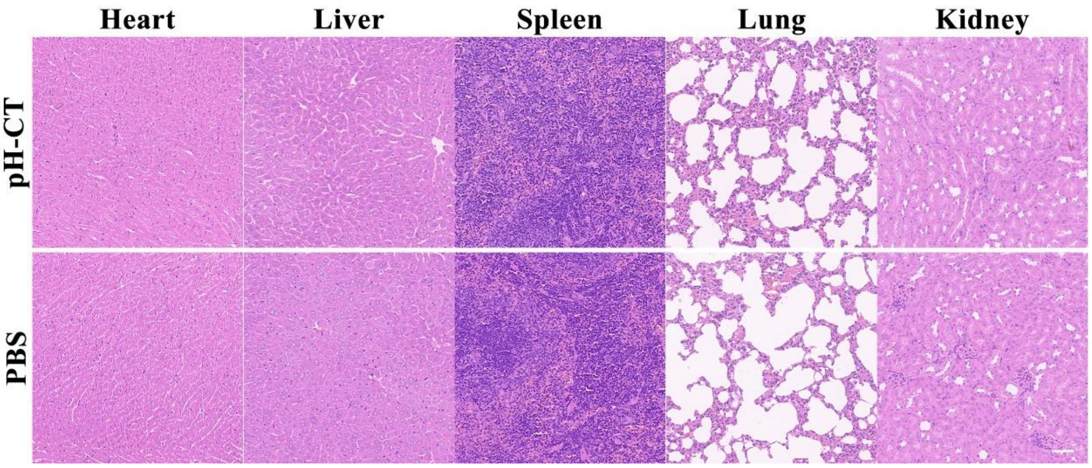

Figure S21. H&E analysis after pH-CT treatment. H&E staining of heart, liver, spleen, lung and kidney slices harvested on the 20th day after treatment. Scale bar: 10 μm.

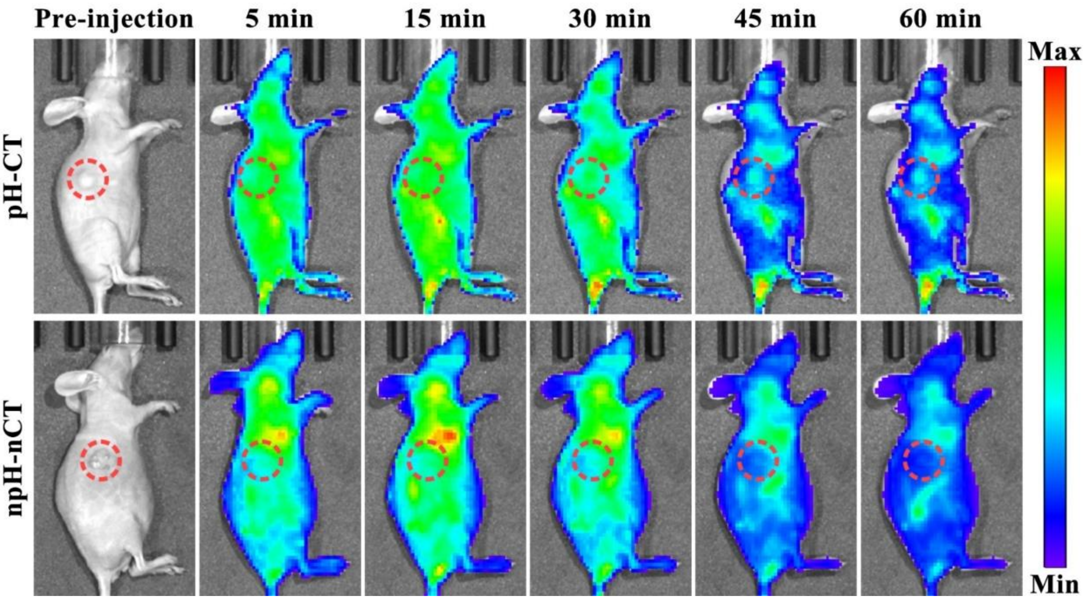

Figure S22. Accurate *in viv*o tumor imaging by pH-CT. *In vivo* time-lapse fluorescence imaging of HepG-2-bearing mice after *i.v.* injection with different probes. Corresponding tumor sites are indicated by red circles.

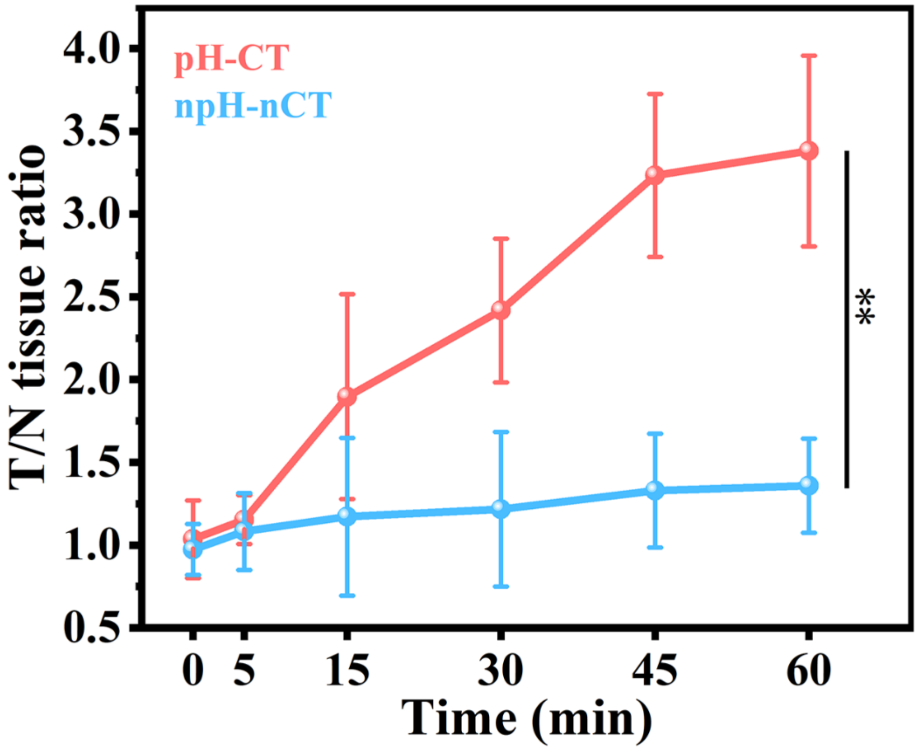

Figure S23. Difference between pH-CT and control probe in T/N ratio. Relative T/N fluorescence intensity ratio as a function of time after various treatments (n=3). Statistical significance was calculated by two-tailed Student's t-test: **, $P < 0.01$.

3. Supplementary Table

Table S1. DNA sequences used in this work.

Name	DNA sequences (5'-3')
**Apt-c-Met**	A*T*C*AGGCTGGATGGTAGCTCGGTCGGGGTGGGTGGGTTGGCAAGTCTGATAAACATTCA/iCy3dT/GAGCTAT*A*T*A
**i-motif-Met**	C*C*C*CCCTCCCCCCTATATAGCTCA/iBHQ2dT/GAATCCCCCCTCCC*C*C*C
**Apt-TfR**	G*G*A*TAGGGATTCTGTTGGTCGGCTGGTTGGTATCCTTATATAGCTCA/iCy5dT/GAA*T*G*T
**i-motif-TfR**	C*C*C*CCCTCCCCCCACATTCA/iBHQ2dT/GAGCTATACCCCCCTCCC*C*C*C
**Ctrl-Apt-c-Met**	T*T*T*TTTTTTTTTTTTTTTTTTTTTTTTTTTTTTTTTTTTTTTTTTTTTTTAAACATTCATGAGCTAT*A*T*A
**Ctrl-T-motif-c-Met**	T*T*T*TTTTTTTTTTTATATAGCTCATGAATTTTTTTTTTT*T*T*T
**Ctrl-Apt-TfR**	T*T*T*TTTTTTTTTTTTTTTTTTTTTTTTTTTTTTTTTTATATAGCTCATGAA*T*G*T

**Ctrl-T-motif-Apt-TfR**	T*T*T*TTTTTTTTTTTATATAGCTCATGAATTTTTTTTTTT*T*T*T

Note:

Red letters denote modifications.

Underlined letters denote random sequence.

* denotes phosphorothioate bonds.
